# Supplementary material for: miR‐205 mediates adaptive resistance to MET inhibition via ERRFI1 targeting and raised EGFR signaling
Source: EMBO Mol Med. 2018 Jul 24;10(9):e8746. doi: 10.15252/emmm.201708746 (PMC6127885; doi:10.15252/emmm.201708746)
Supplement: Supplementary file 1 — Appendix [file EMMM-10-e8746-s001.pdf]

miR-205 mediates adaptive resistance to MET inhibition

via ERRFI1 targeting and raised EGFR signaling

Cristina Migliore*[1,2], Elena Morando[2], Elena Ghiso[2], Sergio Anastasi[3], Vera P. Leoni[4],

Maria Apicella[2], Davide Cora'[1,2,5], Anna Sapino[2,6], Filippo Pietrantonio[7,8], Filippo De Braud[7,8],

Amedeo Columbano[4], Oreste Segatto[3]* and Silvia Giordano[1,2]*.

APPENDIX PDF

• **APPENDIX FIGURE LEGENDS**
• ***Appendix Figure S1: KATO II and SNU-5 dose-response curve to test resistance.***
• ***Appendix Figure S2: miR-205 genomic sequence.***
• ***Appendix Figure S3: control of miR-205 expression upon transfection/silencing.***
• ***Appendix Figure S4: control of miR-205 expression in GTL16 wt and R-CRIZ used for in vivo experiments.***
• ***Appendix Figure S5: PTEN was not down-regulated in resistant vs wt cells.***
• ***Appendix Figure S6: Expression (Fold-Change) of genes predicted to be miR-205 targets and showing significant modulation between resistant and wt cells.***
• ***Appendix Figure S7: ERRFI1 overexpression re-sensitized cells to MET inhibition.***
• ***Appendix Figure S8: miR-205 transduction led to ERRFI1 downregulation and EGFR overexpression in not-MET addicted A549 cells.***
• ***Appendix Figure S9: Blocking EGFR activation re-sensitized GTL16 R-CRIZ cells to MET inhibition.***
• ***Appendix Figure S10: miR-205 expression was not consistently up-regulated in EGFR addicted cells rendered resistant to EGFR TKIs.***
• ***Appendix Figure S11: Table exact P-Values for Figures***
• ***Appendix Figure S12: Table exact P-Values for Expanded View Figures***
• ***Appendix Figure S13: Table exact P-Values for Appendix Supplementary Figures***

APPENDIX FIGURE LEGENDS

Appendix Figure S1: KATO II and SNU-5 dose-response curve to test resistance. **(A,B)** KATO II (A) and SNU-5 (B) cells, either parental (wt) or resistant to the indicated MET-TKIs, were exposed to escalating concentrations of the indicated drugs. Cell viability was measured after 72 hours (CellTiterGlo) to derive the indicated IC_{50} values.

Data information: (A,B) P<0.001 one-way-ANOVA, Bonferroni's multiple comparisons test.

Appendix Figure S2: miR-205 genomic sequence. **(A)** Schematic representation of CpGs in the miR-205 genomic locus: grey arrows display location of PCR and sequencing primers (dark and light grey, respectively) used for DNA methylation analysis of the six CpG (black dots) by pyrosequencing. **(B)** Genomic sequence containing miR-205 gene, before and after bisulfite conversion. Analyzed CpGs are highlighted in bold letters.

Appendix Figure S3: control of miR-205 expression upon transfection/silencing. *MiR-205* expression was evaluated by RT-qPCR in EBC-1-R **(A)**, GTL16-R **(B)**, SG16-R **(C)**, EBC-1 wt **(D)**, GTL16 wt **(E)** and SG16 wt **(F)** cells, upon ectopic expression of miR-205 mimic or antagomiR. As shown, miR-205 was over-expressed in wt cells and silenced in resistant cells. n=3 per condition. Data information: (A-F) average + SD. *** P< 0.001, two-tailed t-test.

Appendix Figure S4: control of miR-205 expression in GTL16 wt and R-CRIZ used for in vivo experiments. **(A)** MiR-205 expression was evaluated by RT-qPCR in GTL16 R-CRIZ upon lentiviral transduction with pCDH-ANTI-miR-205 or control vector (pCDH). MiR-205 expression was decreased upon anti-miR-205 infection. **(B)** MiR-205 expression was evaluated by RT-qPCR in GTL16 wt cells upon lentiviral transduction with pCDH -miR-205 or control vector (pCDH). MiR-205 expression was increased upon miR-205 infection. Data information: (A-B) n=3 per condition, average + SD. *** P< 0.001, two-tailed t-test.

Appendix Figure S5: PTEN was not down-regulated in resistant vs wt cells. PTEN expression was evaluated by WB in EBC-1, GTL16 and SG16 wt and resistant (R-) cells. PTEN expression was not reduced in resistant cells compared to wt. Actin was used as loading control.

Appendix Figure S6: Expression (Fold-Change) of genes predicted to be miR-205 targets and showing significant modulation between resistant and wt cells. Predicted miR-205 targets were downloaded from TargetScan human. Differentially expressed genes were defined by | FC| > 2 and FDR < 0.1 (see Methods). For each cell line, the Expression Fold-Change was always computed in term of resistant versus wt expression data.

Appendix Figure S7: ERRFI1 overexpression re-sensitized cells to MET inhibition. **(A**) Western Blot analysis of ERFFI1 expression upon lentiviral transduction in the indicated cell lines. Actin was used as loading control. **(B)** GTL16 resistant cells were transduced with either empty (pCDH) or ERRFI1-encoding (pCDH ERRFI1) recombinant lentivirus stocks. 3000 cells/well were seeded in the presence of MET TKI and viability was evaluated 72h later. Chart shows a significant decrease in cell viability upon ERRFI1 over-expression in cells resistant to Crizotinib (R-CRIZ), but not in those resistant to PHA-665752 (R-PHA). n=4 per condition. Average + SD. *P<0.05, two-tailed t-test.

Appendix Figure S8: miR-205 transduction led to ERRFI1 downregulation and EGFR overexpression in not-MET addicted A549 cells. **(A)** MiR-205 precursor was ectopically expressed in A549 NSCLC cells. MiR-205 expression was evaluated by RT-qPCR. n=3 per condition, average + SD. *** P< 0.001, two-tailed t-test. **(B)** Expression of ERRFI1, pEGFR and EGFR was evaluated by WB in cells shown in (A). Actin was used as loading control. As shown, ERRFI1 was downregulated while EGFR and p-EGFR were upregulated upon miR-205 over-expression.

Appendix Figure S9: Blocking EGFR activation re-sensitized GTL16 R-CRIZ cells to MET inhibition. GTL16 wt and resistant cells were seeded (3000 cells/well) in a 96 well costar plate and kept in normal growth conditions (in the absence (wt) or in the presence (R-) of MET-TKI). Afatinib was added at the indicated doses. Viability was assessed 72h later (CellTiter Glo). n=4 per condition. Average + SD. *** P< 0.001; **P<0.01; *P<0.05, two-way ANOVA Bonferroni's multiple comparisons.

Appendix Figure S10: miR-205 expression was not consistently up-regulated in EGFR addicted cells rendered resistant to EGFR TKIs. Whole miRNA expression was evaluated by Taqman-Low-Density-Array (TLDA) in EGFR addicted lung adenocarcinoma cells (HCC4006, PC-9, HCC827 and H3255) rendered resistant to first generation inhibitors (erlotinib, gefitinib) and second generation inhibitors (afatinib, AZD-8931, dacomitinib). MiR-205 expression was normalized to RNU48. ERL = erlotinib, GEF = gefitinib, AFA = afatinib, AZD = AZD-8931, DACO = dacomitinib.

APPENDIX FIGURE S1

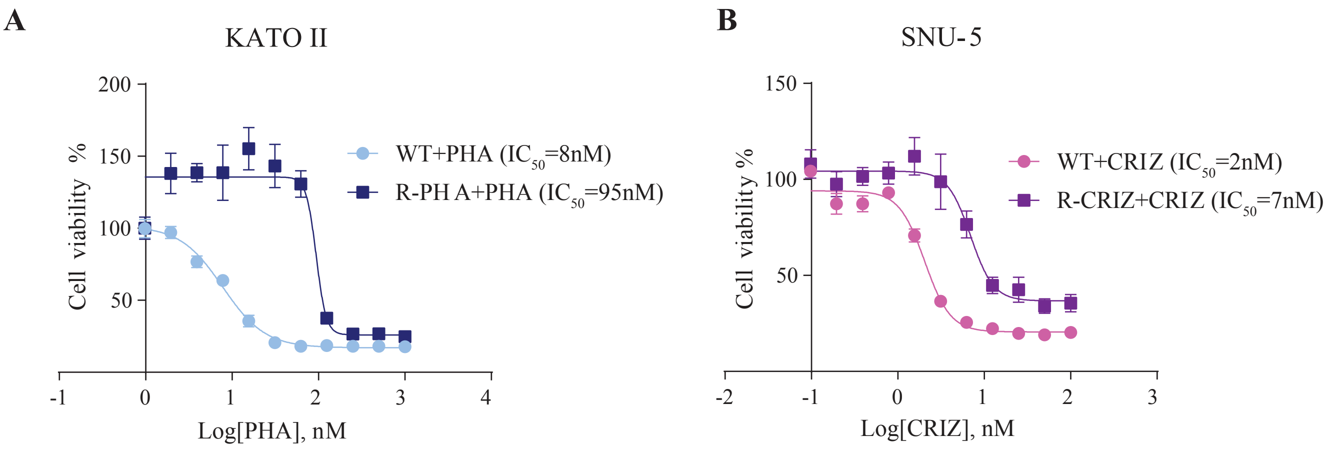

APPENDIX FIGURE S2

A

Chr1: 209,432,133 - 209,432,403

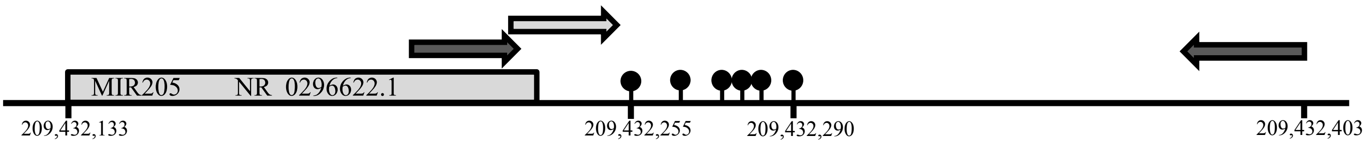

B

GENOMIC SEQUENCE

GGCCCCACAGACCCACCTCCCCAACTCAGCACTGTCTGTCTGTGCAGCAGGTGCAAGGAC
GTGTTGAACTAGCTCTCTGCAGCCTCCTTGGAGGATGTGATCCTATGGGAGGGGTAGGAG
TATTCAGGTCCTTGACATCTCCCAAATGTGTGATTCCGGGATGCCAAAGGCCTTTGGCCA
GGTAATGCAGTGTCTACAGGCTGAGGTTGACATGCATCCCCACCCTCTGAGAAAAAGATC
CTCAGACAATCCATGTGCTTCTCTTGTCCTTCATTCCACCGGAGTCTGTCTCATACCCAA
CCAGATTTCAGTGGAGTGAAGTTCAGGAGGCATGGAGCTGACAACCATGAGGCCT**CG**GCA
GCCAC**CG**CCACCAC**CG**C**CG**C**CG**CCACCAC**CG**TAGCAGCAGCAGCAGCAGCAGCAGCA
GCAGCAGCAGCAAGAGTAACTCTGACTTAGGAATAGAGACAGCCAGAGAGAAATGTGATC
AATGAAGGAGACATCTGGAGTGTGCGTGCTTCTTCAGAGGGACGGGTGATGGGCAGATTG

GENOMIC SEQUENCE OBTAINED UPON BISULFITE CONVERSION

GGTTTTATAGATTTATTTTTTTAATTTAGTATTGTTTGTTTGTGTAGTAGGTGTAAGGAT
GTGTTGAATTAGTTTTTTGTAGTTTTTTTGGAGGATGTGATTTTATGGGAGGGGTAGGAG
TATTTAGGTTTTTGATATTTTTTAAATGTGTGATTTCGGGATGTTAAAGGTTTTTGGTTA
GGTAATGTAGTGTTTATAGGTTGAGGTTGATATGTATTTTTATTTTTTGAGAAAAAGATT
TTTAGATAATTTATGTGTTTTTTTTGTTTTTTATTTTATCGGAGTTTGTTTTATATTTAA
TTAGATTTTAG**TGGAGTGAAGTTTAGGAGGTATGG****AGTTGATAATTATGAGGTT**T**CG**GTA
GTTAT**CG**TTATTAT**CG**T**CG**T**CG**TTATTAT**CG**TAGTAGTAGTAGTAGTAGTAGTAGTA
GTAGTAGTAGTAAGAGTAATTTTGATTTAGGAATAGAGATAGTTAGAGAGAAATGTGATT
AATGAAGGAGATATTTGGAGTGTGCGTGTTTTTTTAGAGGGACGGGTGATGGGTAGATTG

LEGEND:

NR_029622.1 = Homo sapiens microRNA 205 (MIR205)

PCR PRIMERS:

Biotinylated Reverse: **CACACTCCAAATATCTCCTTCATT**
Forward: **TGGAGTGAAGTTTAGGAGGTATGG**

SEQUENCING PRIMER :

CpGmiR205 TSS S1: **GAGTTGATAATTATGAGGTT**

CG: CpGs in the sequence

CG: CpGs evaluated

APPENDIX FIGURE S3

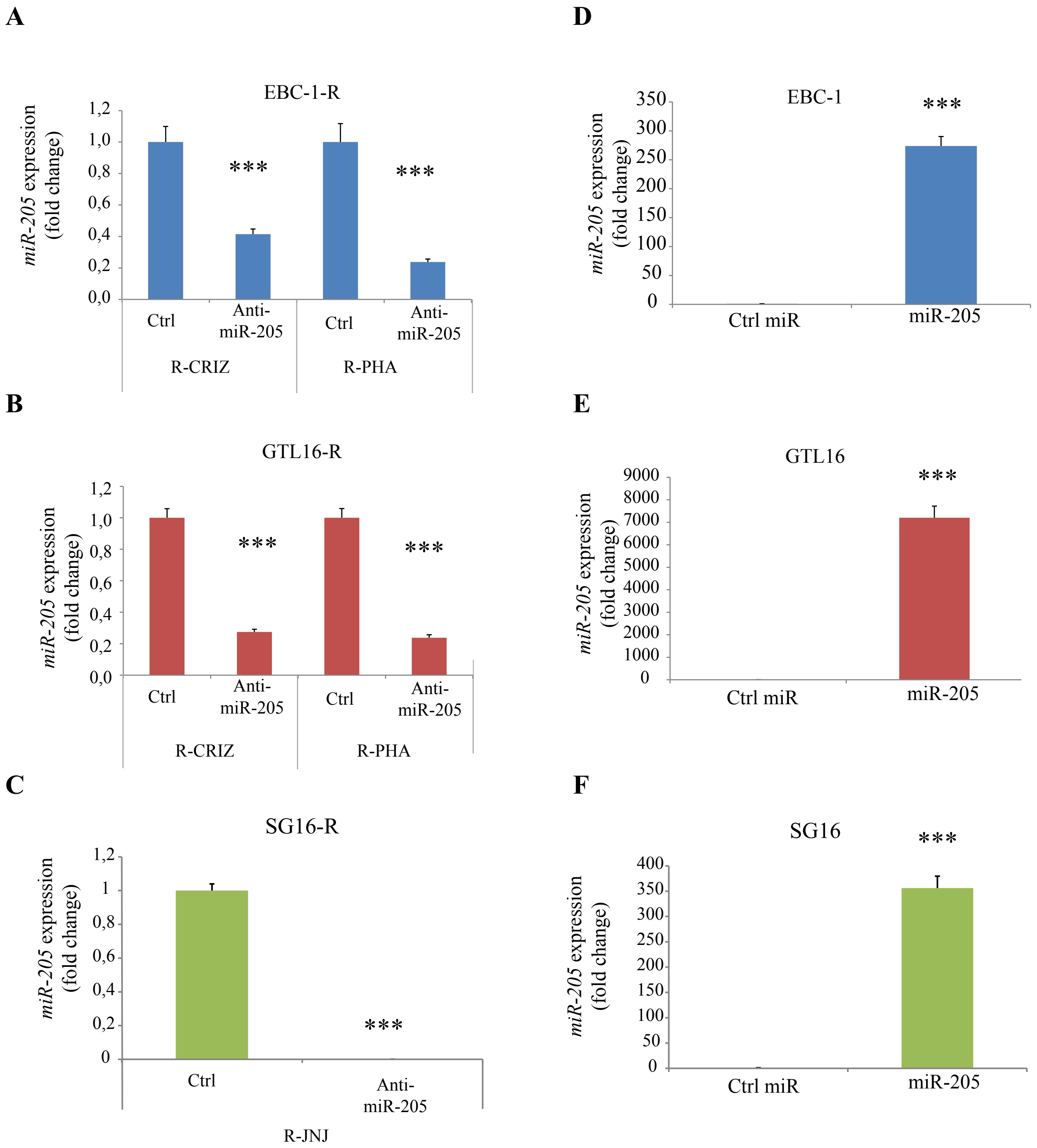

APPENDIX FIGURE S4

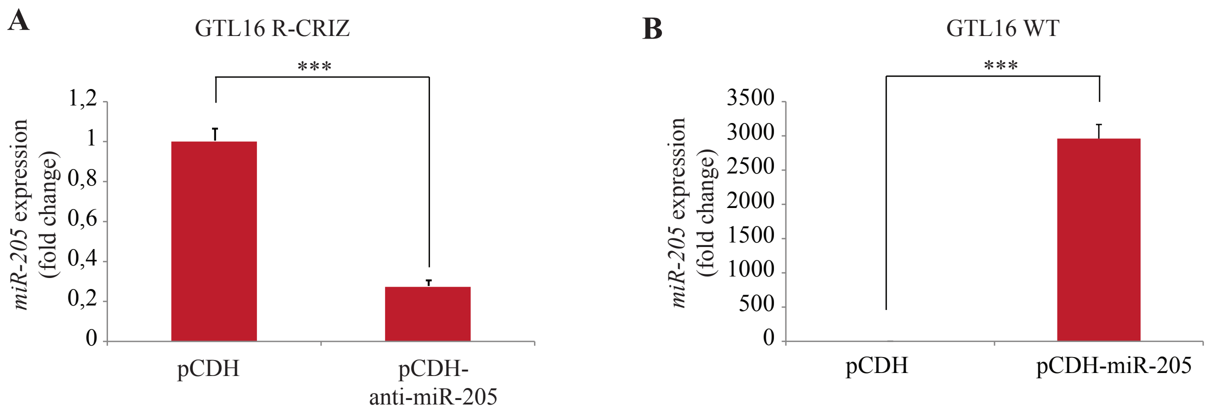

APPENDIX FIGURE S5

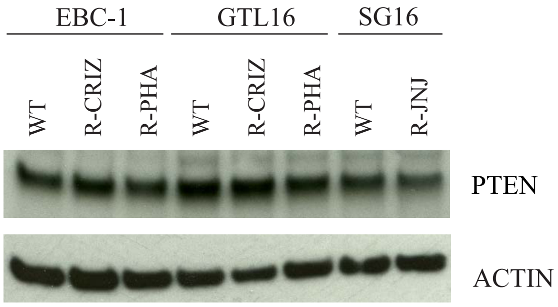

APPENDIX FIGURE S6

EBC-1-R-CRIZ *vs* EBC-1 wt				GTL16-R-CRIZ *vs* GTL16 wt			
UP-REGULATED GENES		DOWN-REGULATED GENES		UP-REGULATED GENES		DOWN-REGULATED GENES	
**Gene_id**	**Fold_change**	**Gene_id**	**Fold_change**	**Gene_id**	**Fold_change**	**Gene_id**	**Fold_change**
PHYHIPL	104,17	NSF	-2,02	NRCAM	5,52	ERRFI1	-2,04
NRCAM	77,78	NECAP1	-2,05	GATA3	4,40	CADM1	-2,11
XYLT1	34,10	KPNA1	-2,11	DLG2	4,09	MXD1	-2,12
ADAMTS9	32,97	EPB41L4B	-2,12	ADAMTS9	3,48	APBB2	-2,15
LRP4	23,51	RNF213	-2,18	KLF12	3,41	HOXC12	-2,25
PTPRG	20,97	NPEPPS	-2,24	PLCB1	3,22	AAK1	-2,33
LIMS2	15,44	PAQR5	-2,28	P2RY1	2,90	IRF1	-2,34
MAGI2	13,90	MMD	-2,34	CRMP1	2,63	LAMC1	-2,49
KIF26B	10,96	EPB41	-2,41	DLEU1	2,57	RPS6KA3	-2,60
CDK14	10,50	LPCAT1	-2,42	SYPL2	2,54	SEPT5	-2,61
PDE3B	10,28	WDTC1	-2,45	ARHGAP24	2,49	TSC22D1	-2,98
SULF1	9,85	AGPAT6	-2,46	DNM3	2,30	KIF1B	-3,00
BICC1	9,01	RARA	-2,53	EFCAB4A	2,16	RBMS1	-3,15
GBA	7,10	TFDP2	-2,53	PAQR5	2,12	BAMBI	-3,45
PRDM16	6,85	HS3ST1	-2,57	DOK4	2,10	NEU1	-4,06
BAMBI	6,44	RTN3	-2,60	ERBB3	2,03	C20orf194	-4,09
THBS1	5,84	UBE2E3	-2,76	LRP4	2,00	KIF26B	-4,70
BMF	5,11	C16orf52	-2,76			EREG	-4,90
TP53INP1	4,18	PI16	-2,95			THBS1	-5,05
NCOA1	3,99	ENC1	-3,14			WWC3	-5,88
PHLDA3	3,88	DOCK3	-3,18			PROX1	-7,67
TBX3	3,73	SEMA7A	-3,18			LSAMP	-8,25
INSR	3,63	TMEM144	-3,23			ZEB1	-9,47
KLF7	3,36	PRKCA	-3,36			KCNMB4	-19,33
LPAR1	3,17	ABHD10	-3,42				
STON2	3,15	MICAL2	-3,51				
EMP2	3,07	SORBS1	-3,58				
ITGA5	2,96	VWA7	-3,83				
LDLRAD3	2,91	CHN1	-3,99				
SEPT5	2,81	PHC2	-4,06				
LAMC1	2,76	VEGFA	-4,33				
CTH	2,67	ARHGAP24	-4,84				
DUSP7	2,43	MAGI1	-5,03				
PAX9	2,34	SPRY1	-5,23				
LRP1	2,30	SOGA3	-5,38				
TUBB6	2,22	SUSD1	-6,31				
		PTPRJ	-6,98				
		EREG	-7,11				
		AL031666.2	-9,29				
		ERRFI1	-12,60				
		ENPP4	-15,99				
		EFCAB4A	-17,51				
		ZEB1	-19,45				
		IL1RAPL1	-43,22				

APPENDIX FIGURE S7

A

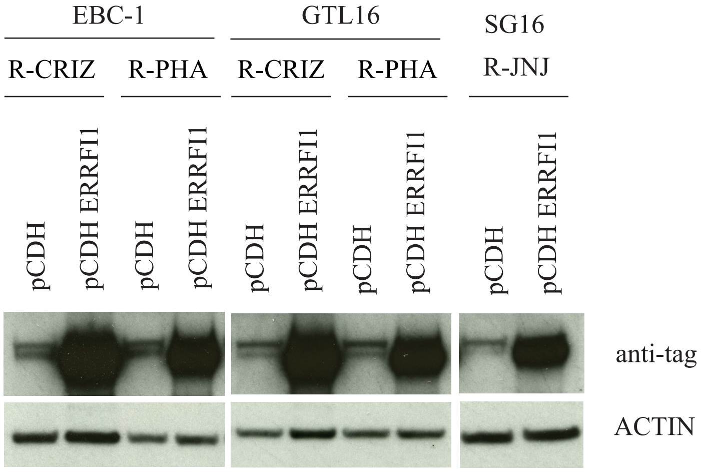

B

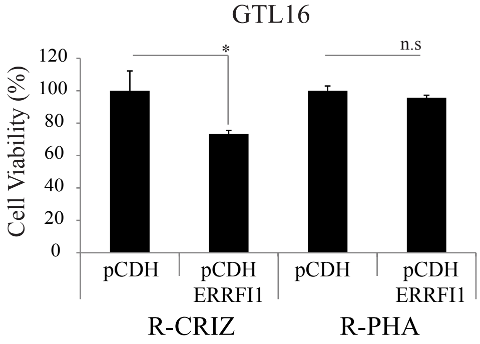

APPENDIX FIGURE S8

A

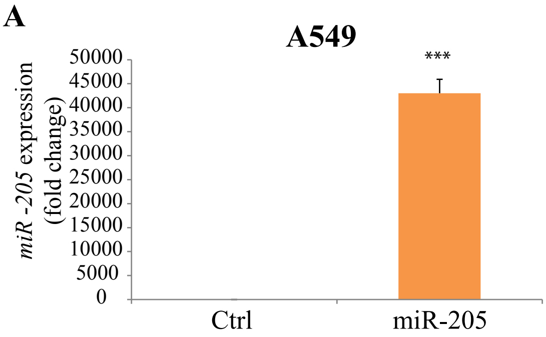

B

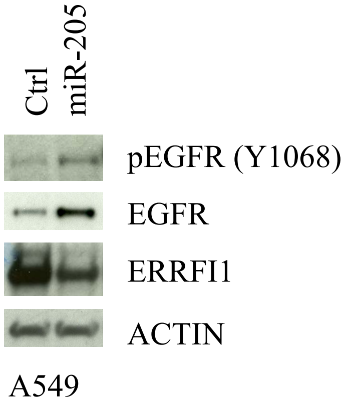

APPENDIX FIGURE S9

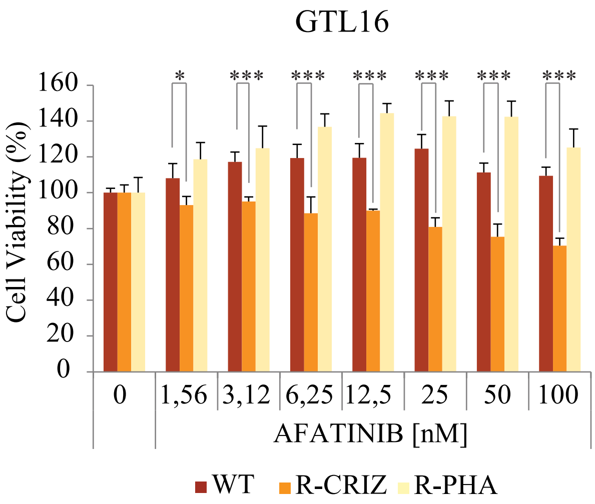

APPENDIX FIGURE S10

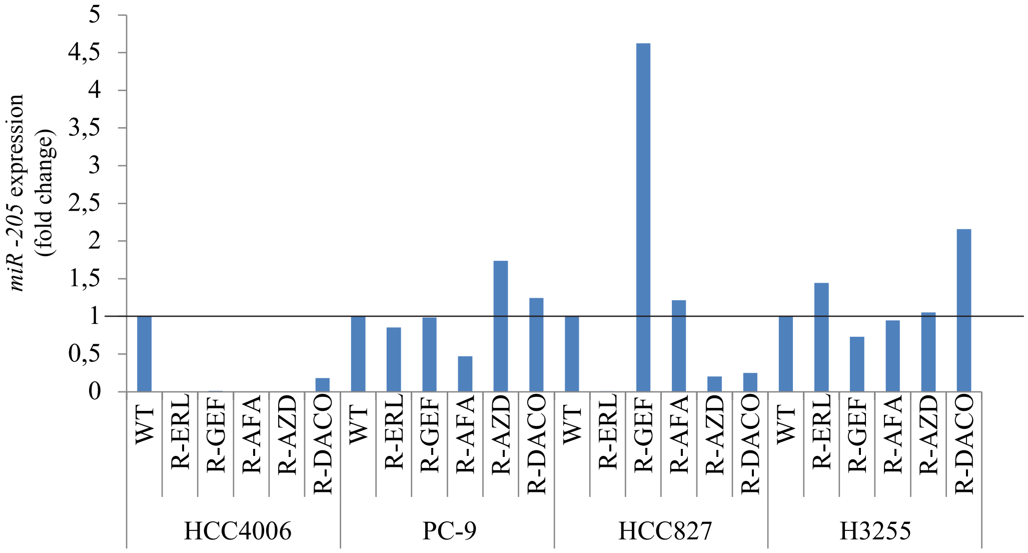

Appendix Figure S11

Figure	Panel	experiment	n-value	p-value	Significativity	TEST
**1**	A	EBC-1 WT+CRIZ vs EBC-1 R-CRIZ + CRIZ (25nM)	3	<0.0001	***	Two-way ANOVA
		EBC-1 WT+CRIZ vs EBC-1 R-CRIZ + PHA (25nM)	3	<0.0001	***	Two-way ANOVA
		EBC-1 WT+CRIZ vs EBC-1 R-CRIZ + JNJ(25nM)	3	<0.0001	***	Two-way ANOVA
		EBC-1 WT + PHA vs. EBC-1 R-PHA + CRIZ(25nM)	3	<0.0001	***	Two-way ANOVA
		EBC-1 WT + PHA vs. EBC-1 R-PHA + PHA(25nM)	3	<0.0001	***	Two-way ANOVA
		EBC-1 WT + PHA vs. EBC-1 R-PHA + JNJ(25nM)	3	<0.0001	***	Two-way ANOVA
		EBC-1 R-CRIZ + CRIZ vs. EBC-1 R-CRIZ + PHA(25nM)	3	>0,9999	ns	Two-way ANOVA
		EBC-1 R-CRIZ + CRIZ vs. EBC-1 R-CRIZ + JNJ(25nM)	3	>0,9999	ns	Two-way ANOVA
		EBC-1 R-CRIZ + CRIZ vs. EBC-1 R-PHA + CRIZ(25nM)	3	>0,9999	ns	Two-way ANOVA
		EBC-1 R-CRIZ + CRIZ vs. EBC-1 R-PHA + PHA(25nM)	3	>0,9999	ns	Two-way ANOVA
		EBC-1 R-CRIZ + CRIZ vs. EBC-1 R-PHA + JNJ(25nM)	3	>0,9999	ns	Two-way ANOVA
		EBC-1 R-CRIZ + PHA vs. EBC-1 R-CRIZ + JNJ(25nM)	3	>0,9999	ns	Two-way ANOVA
		EBC-1 R-CRIZ + PHA vs. EBC-1 R-PHA + CRIZ(25nM)	3	>0,9999	ns	Two-way ANOVA
		EBC-1 R-CRIZ + PHA vs. EBC-1 R-PHA + PHA(25nM)	3	>0,9999	ns	Two-way ANOVA
		EBC-1 R-CRIZ + PHA vs. EBC-1 R-PHA + JNJ(25nM)	3	>0,9999	ns	Two-way ANOVA
		EBC-1 R-CRIZ + JNJ vs. EBC-1 R-PHA + CRIZ(25nM)	3	>0,9999	ns	Two-way ANOVA
		EBC-1 R-CRIZ + JNJ vs. EBC-1 R-PHA + PHA(25nM)	3	>0,9999	ns	Two-way

Appendix Figure S11

					ANOVA
	EBC-1 R-CRIZ + JNJ vs. EBC-1 R-PHA + JNJ(25nM)	3	>0,9999	ns	Two-way ANOVA
	EBC-1 R-PHA + CRIZ vs. EBC-1 R-PHA + PHA(25nM)	3	>0,9999	ns	Two-way ANOVA
	EBC-1 R-PHA + CRIZ vs. EBC-1 R-PHA + JNJ(25nM)	3	>0,9999	ns	Two-way ANOVA
	EBC-1 R-PHA + PHA vs. EBC-1 R-PHA + JNJ(25nM)	3	>0,9999	ns	Two-way ANOVA
	EBC-1 WT+CRIZ vs. EBC-1 R-CRIZ + CRIZ (50nM)	3	<0.0001	***	Two-way ANOVA
	EBC-1 WT+CRIZ vs. EBC-1 R-CRIZ + PHA(50nM)	3	<0.0001	***	Two-way ANOVA
	EBC-1 WT+CRIZ vs. EBC-1 R-CRIZ + JNJ(50nM)	3	<0.0001	***	Two-way ANOVA
	EBC-1 WT + PHA vs. EBC-1 R-PHA + CRIZ(50nM)	3	<0.0001	***	Two-way ANOVA
	EBC-1 WT + PHA vs. EBC-1 R-PHA + PHA(50nM)	3	<0.0001	***	Two-way ANOVA
	EBC-1 WT + PHA vs. EBC-1 R-PHA + JNJ(50nM)	3	<0.0001	***	Two-way ANOVA
	EBC-1 R-CRIZ + CRIZ vs. EBC-1 R-CRIZ + PHA(50nM)	3	>0,9999	ns	Two-way ANOVA
	EBC-1 R-CRIZ + CRIZ vs. EBC-1 R-CRIZ + JNJ(50nM)	3	>0,9999	ns	Two-way ANOVA
	EBC-1 R-CRIZ + CRIZ vs. EBC-1 R-PHA + CRIZ(50nM)	3	>0,9999	ns	Two-way ANOVA
	EBC-1 R-CRIZ + CRIZ vs. EBC-1 R-PHA + PHA(50nM)	3	>0,9999	ns	Two-way ANOVA
	EBC-1 R-CRIZ + CRIZ vs. EBC-1 R-PHA + JNJ(50nM)	3	>0,9999	ns	Two-way ANOVA
	EBC-1 R-CRIZ + PHA vs. EBC-1 R-CRIZ + JNJ(50nM)	3	>0,9999	ns	Two-way ANOVA
	EBC-1 R-CRIZ + PHA vs. EBC-1 R-PHA + CRIZ(50nM)	3	>0,9999	ns	Two-way ANOVA
	EBC-1 R-CRIZ + PHA vs. EBC-1 R-	3	>0,9999	ns	Two-

	PHA + PHA(50nM)				way ANOVA
	EBC-1 R-CRIZ + PHA vs. EBC-1 R-PHA + JNJ(50nM)	3	>0,9999	ns	Two-way ANOVA
	EBC-1 R-CRIZ + JNJ vs. EBC-1 R-PHA + CRIZ(50nM)	3	>0,9999	ns	Two-way ANOVA
	EBC-1 R-CRIZ + JNJ vs. EBC-1 R-PHA + PHA(50nM)	3	>0,9999	ns	Two-way ANOVA
	EBC-1 R-CRIZ + JNJ vs. EBC-1 R-PHA + JNJ(50nM)	3	>0,9999	ns	Two-way ANOVA
	EBC-1 R-PHA + CRIZ vs. EBC-1 R-PHA + PHA(50nM)	3	>0,9999	ns	Two-way ANOVA
	EBC-1 R-PHA + CRIZ vs. EBC-1 R-PHA + JNJ(50nM)	3	>0,9999	ns	Two-way ANOVA
	EBC-1 R-PHA + PHA vs. EBC-1 R-PHA + JNJ(50nM)	3	>0,9999	ns	Two-way ANOVA
	EBC-1 WT+CRIZ vs. EBC-1 R-CRIZ + CRIZ (100nM)	3	<0.0001	***	Two-way ANOVA
	EBC-1 WT+CRIZ vs. EBC-1 R-CRIZ + PHA(100nM)	3	<0.0001	***	Two-way ANOVA
	EBC-1 WT+CRIZ vs. EBC-1 R-CRIZ + JNJ(100nM)	3	<0.0001	***	Two-way ANOVA
	EBC-1 R-CRIZ + CRIZ vs. EBC-1 R-CRIZ + PHA(100nM)	3	>0,9999	ns	Two-way ANOVA
	EBC-1 R-CRIZ + CRIZ vs. EBC-1 R-CRIZ + JNJ(100nM)	3	>0,9999	ns	Two-way ANOVA
	EBC-1 R-CRIZ + CRIZ vs. EBC-1 R-PHA + CRIZ(100nM)	3	>0,9999	ns	Two-way ANOVA
	EBC-1 R-CRIZ + CRIZ vs. EBC-1 R-PHA + PHA(100nM)	3	>0,9999	ns	Two-way ANOVA
	EBC-1 R-CRIZ + CRIZ vs. EBC-1 R-PHA + JNJ(100nM)	3	>0,9999	ns	Two-way ANOVA
	EBC-1 R-CRIZ + PHA vs. EBC-1 R-CRIZ + JNJ(100nM)	3	>0,9999	ns	Two-way ANOVA
	EBC-1 R-CRIZ + PHA vs. EBC-1 R-PHA + CRIZ(100nM)	3	>0,9999	ns	Two-way ANOVA

Appendix Figure S11

	EBC-1 R-CRIZ + PHA vs. EBC-1 R-PHA + PHA(100nM)	3	>0,9999	ns	Two-way ANOVA
	EBC-1 R-CRIZ + PHA vs. EBC-1 R-PHA + JNJ(100nM)	3	>0,9999	ns	Two-way ANOVA
	EBC-1 R-CRIZ + JNJ vs. EBC-1 R-PHA + CRIZ(100nM)	3	>0,9999	ns	Two-way ANOVA
	EBC-1 R-CRIZ + JNJ vs. EBC-1 R-PHA + PHA(100nM)	3	>0,9999	ns	Two-way ANOVA
	EBC-1 R-CRIZ + JNJ vs. EBC-1 R-PHA + JNJ(100nM)	3	>0,9999	ns	Two-way ANOVA
	EBC-1 R-PHA + CRIZ vs. EBC-1 R-PHA + PHA(100nM)	3	>0,9999	ns	Two-way ANOVA
	EBC-1 R-PHA + CRIZ vs. EBC-1 R-PHA + JNJ(100nM)	3	>0,9999	ns	Two-way ANOVA
	EBC-1 R-PHA + PHA vs. EBC-1 R-PHA + JNJ(100nM)	3	>0,9999	ns	Two-way ANOVA
	EBC-1 WT+CRIZ vs. EBC-1 R-CRIZ + CRIZ (200nM)	3	<0.0001	***	Two-way ANOVA
	EBC-1 WT+CRIZ vs. EBC-1 R-CRIZ + PHA(200nM)	3	<0.0001	***	Two-way ANOVA
	EBC-1 WT+CRIZ vs. EBC-1 R-CRIZ + JNJ(200nM)	3	<0.0001	***	Two-way ANOVA
	EBC-1 WT + PHA vs. EBC-1 R-PHA + CRIZ(200nM)	3	<0.0001	***	Two-way ANOVA
	EBC-1 WT + PHA vs. EBC-1 R-PHA + PHA(200nM)	3	<0.0001	***	Two-way ANOVA
	EBC-1 WT + PHA vs. EBC-1 R-PHA + JNJ(200nM)	3	<0.0001	***	Two-way ANOVA
	EBC-1 R-CRIZ + CRIZ vs. EBC-1 R-CRIZ + PHA(200nM)	3	>0,9999	ns	Two-way ANOVA
	EBC-1 R-CRIZ + CRIZ vs. EBC-1 R-CRIZ + JNJ(200nM)	3	>0,9999	ns	Two-way ANOVA
	EBC-1 R-CRIZ + CRIZ vs. EBC-1 R-PHA + CRIZ(200nM)	3	>0,9999	ns	Two-way ANOVA
	EBC-1 R-CRIZ + CRIZ vs. EBC-1 R-PHA + PHA(200nM)	3	>0,9999	ns	Two-way

					ANOVA
	EBC-1 R-CRIZ + CRIZ vs. EBC-1 R-PHA + JNJ(200nM)	3	>0,9999	ns	Two-way ANOVA
	EBC-1 R-CRIZ + PHA vs. EBC-1 R-CRIZ + JNJ(200nM)	3	>0,9999	ns	Two-way ANOVA
	EBC-1 R-CRIZ + PHA vs. EBC-1 R-PHA + CRIZ(200nM)	3	>0,9999	ns	Two-way ANOVA
	EBC-1 R-CRIZ + PHA vs. EBC-1 R-PHA + PHA(200nM)	3	>0,9999	ns	Two-way ANOVA
	EBC-1 R-CRIZ + PHA vs. EBC-1 R-PHA + JNJ(200nM)	3	>0,9999	ns	Two-way ANOVA
	EBC-1 R-CRIZ + JNJ vs. EBC-1 R-PHA + CRIZ(200nM)	3	>0,9999	ns	Two-way ANOVA
	EBC-1 R-CRIZ + JNJ vs. EBC-1 R-PHA + PHA(200nM)	3	>0,9999	ns	Two-way ANOVA
	EBC-1 R-CRIZ + JNJ vs. EBC-1 R-PHA + JNJ(200nM)	3	>0,9999	ns	Two-way ANOVA
	EBC-1 R-PHA + CRIZ vs. EBC-1 R-PHA + PHA(200nM)	3	>0,9999	ns	Two-way ANOVA
	EBC-1 R-PHA + CRIZ vs. EBC-1 R-PHA + JNJ(200nM)	3	>0,9999	ns	Two-way ANOVA
	EBC-1 R-PHA + PHA vs. EBC-1 R-PHA + JNJ(200nM)	3	>0,9999	ns	Two-way ANOVA
	EBC-1 WT+CRIZ vs. EBC-1 R-CRIZ + CRIZ(400nM)	3	<0.0001	***	Two-way ANOVA
	EBC-1 WT+CRIZ vs. EBC-1 R-CRIZ + PHA(400nM)	3	<0.0001	***	Two-way ANOVA
	EBC-1 WT+CRIZ vs. EBC-1 R-CRIZ + JNJ(400nM)	3	<0.0001	***	Two-way ANOVA
	EBC-1 WT + PHA vs. EBC-1 R-PHA + CRIZ(400nM)	3	<0.0001	***	Two-way ANOVA
	EBC-1 WT + PHA vs. EBC-1 R-PHA + PHA(400nM)	3	<0.0001	***	Two-way ANOVA
	EBC-1 WT + PHA vs. EBC-1 R-PHA + JNJ(400nM)	3	<0.0001	***	Two-way ANOVA
	EBC-1 R-CRIZ + CRIZ vs. EBC-1 R-	3	>0,9999	ns	Two-

Appendix Figure S11

	CRIZ + PHA(400nM)				way ANOVA
	EBC-1 R-CRIZ + CRIZ vs. EBC-1 R-CRIZ + JNJ(400nM)	3	>0,9999	ns	Two-way ANOVA
	EBC-1 R-CRIZ + CRIZ vs. EBC-1 R-PHA + CRIZ(400nM)	3	>0,9999	ns	Two-way ANOVA
	EBC-1 R-CRIZ + CRIZ vs. EBC-1 R-PHA + PHA(400nM)	3	>0,9999	ns	Two-way ANOVA
	EBC-1 R-CRIZ + CRIZ vs. EBC-1 R-PHA + JNJ(400nM)	3	>0,9999	ns	Two-way ANOVA
	EBC-1 R-CRIZ + PHA vs. EBC-1 R-CRIZ + JNJ(400nM)	3	>0,9999	ns	Two-way ANOVA
	EBC-1 R-CRIZ + PHA vs. EBC-1 R-PHA + CRIZ(400nM)	3	>0,9999	ns	Two-way ANOVA
	EBC-1 R-CRIZ + PHA vs. EBC-1 R-PHA + PHA(400nM)	3	>0,9999	ns	Two-way ANOVA
	EBC-1 R-CRIZ + PHA vs. EBC-1 R-PHA + JNJ(400nM)	3	>0,9999	ns	Two-way ANOVA
	EBC-1 R-CRIZ + JNJ vs. EBC-1 R-PHA + CRIZ(400nM)	3	>0,9999	ns	Two-way ANOVA
	EBC-1 R-CRIZ + JNJ vs. EBC-1 R-PHA + PHA(400nM)	3	>0,9999	ns	Two-way ANOVA
	EBC-1 R-CRIZ + JNJ vs. EBC-1 R-PHA + JNJ(400nM)	3	>0,9999	ns	Two-way ANOVA
	EBC-1 R-PHA + CRIZ vs. EBC-1 R-PHA + PHA(400nM)	3	>0,9999	ns	Two-way ANOVA
	EBC-1 R-PHA + CRIZ vs. EBC-1 R-PHA + JNJ(400nM)	3	>0,9999	ns	Two-way ANOVA
	EBC-1 R-PHA + PHA vs. EBC-1 R-PHA + JNJ(400nM)	3	>0,9999	ns	Two-way ANOVA
	EBC-1 WT+CRIZ vs. EBC-1 R-CRIZ + CRIZ(800nM)	3	<0.0001	***	Two-way ANOVA
	EBC-1 WT+CRIZ vs. EBC-1 R-CRIZ + PHA(800nM)	3	<0.0001	***	Two-way ANOVA
	EBC-1 WT+CRIZ vs. EBC-1 R-CRIZ + JNJ(800nM)	3	<0.0001	***	Two-way ANOVA

Appendix Figure S11

	EBC-1 WT + PHA vs. EBC-1 R-PHA + CRIZ(800nM)	3	<0.0001	***	Two-way ANOVA
	EBC-1 WT + PHA vs. EBC-1 R-PHA + PHA(800nM)	3	<0.0001	***	Two-way ANOVA
	EBC-1 WT + PHA vs. EBC-1 R-PHA + JNJ(800nM)	3	<0.0001	***	Two-way ANOVA
	EBC-1 R-CRIZ + CRIZ vs. EBC-1 R-CRIZ + PHA(800nM)	3	>0,9999	ns	Two-way ANOVA
	EBC-1 R-CRIZ + CRIZ vs. EBC-1 R-CRIZ + JNJ(800nM)	3	>0,9999	ns	Two-way ANOVA
	EBC-1 R-CRIZ + CRIZ vs. EBC-1 R-PHA + CRIZ(800nM)	3	>0,9999	ns	Two-way ANOVA
	EBC-1 R-CRIZ + CRIZ vs. EBC-1 R-PHA + PHA(800nM)	3	>0,9999	ns	Two-way ANOVA
	EBC-1 R-CRIZ + CRIZ vs. EBC-1 R-PHA + JNJ(800nM)	3	>0,9999	ns	Two-way ANOVA
	EBC-1 R-CRIZ + PHA vs. EBC-1 R-CRIZ + JNJ(800nM)	3	>0,9999	ns	Two-way ANOVA
	EBC-1 R-CRIZ + PHA vs. EBC-1 R-PHA + CRIZ(800nM)	3	>0,9999	ns	Two-way ANOVA
	EBC-1 R-CRIZ + PHA vs. EBC-1 R-PHA + PHA(800nM)	3	>0,9999	ns	Two-way ANOVA
	EBC-1 R-CRIZ + PHA vs. EBC-1 R-PHA + JNJ(800nM)	3	>0,9999	ns	Two-way ANOVA
	EBC-1 R-CRIZ + JNJ vs. EBC-1 R-PHA + CRIZ(800nM)	3	>0,9999	ns	Two-way ANOVA
	EBC-1 R-CRIZ + JNJ vs. EBC-1 R-PHA + PHA(800nM)	3	>0,9999	ns	Two-way ANOVA
	EBC-1 R-CRIZ + JNJ vs. EBC-1 R-PHA + JNJ(800nM)	3	>0,9999	ns	Two-way ANOVA
	EBC-1 R-PHA + CRIZ vs. EBC-1 R-PHA + PHA(800nM)	3	>0,9999	ns	Two-way ANOVA
	EBC-1 R-PHA + CRIZ vs. EBC-1 R-PHA + JNJ(800nM)	3	>0,9999	ns	Two-way ANOVA
	EBC-1 R-PHA + PHA vs. EBC-1 R-PHA + JNJ(800nM)	3	>0,9999	ns	Two-way

Appendix Figure S11

					ANOVA
	EBC-1 WT+CRIZ vs. EBC-1 R-CRIZ + CRIZ(1600nM)	3	<0.0001	***	Two-way ANOVA
	EBC-1 WT+CRIZ vs. EBC-1 R-CRIZ + PHA(1600nM)	3	<0.0001	***	Two-way ANOVA
	EBC-1 WT+CRIZ vs. EBC-1 R-CRIZ + JNJ(1600nM)	3	<0.0001	***	Two-way ANOVA
	EBC-1 WT + PHA vs. EBC-1 R-PHA + CRIZ(1600nM)	3	<0.0001	***	Two-way ANOVA
	EBC-1 WT + PHA vs. EBC-1 R-PHA + PHA(1600nM)	3	<0.0001	***	Two-way ANOVA
	EBC-1 WT + PHA vs. EBC-1 R-PHA + JNJ(1600nM)	3	<0.0001	***	Two-way ANOVA
	EBC-1 R-CRIZ + CRIZ vs. EBC-1 R-CRIZ + PHA(1600nM)	3	0,0541	ns	Two-way ANOVA
	EBC-1 R-CRIZ + CRIZ vs. EBC-1 R-CRIZ + JNJ(1600nM)	3	0,0014	**	Two-way ANOVA
	EBC-1 R-CRIZ + CRIZ vs. EBC-1 R-PHA + CRIZ(1600nM)	3	>0,9999	ns	Two-way ANOVA
	EBC-1 R-CRIZ + CRIZ vs. EBC-1 R-PHA + PHA(1600nM)	3	0,1558	ns	Two-way ANOVA
	EBC-1 R-CRIZ + CRIZ vs. EBC-1 R-PHA + JNJ(1600nM)	3	0,0084	**	Two-way ANOVA
	EBC-1 R-CRIZ + PHA vs. EBC-1 R-CRIZ + JNJ(1600nM)	3	>0,9999	ns	Two-way ANOVA
	EBC-1 R-CRIZ + PHA vs. EBC-1 R-PHA + CRIZ(1600nM)	3	>0,9999	ns	Two-way ANOVA
	EBC-1 R-CRIZ + PHA vs. EBC-1 R-PHA + PHA(1600nM)	3	>0,9999	ns	Two-way ANOVA
	EBC-1 R-CRIZ + PHA vs. EBC-1 R-PHA + JNJ(1600nM)	3	>0,9999	ns	Two-way ANOVA
	EBC-1 R-CRIZ + JNJ vs. EBC-1 R-PHA + CRIZ(1600nM)	3	0,6473	ns	Two-way ANOVA
	EBC-1 R-CRIZ + JNJ vs. EBC-1 R-PHA + PHA(1600nM)	3	>0,9999	ns	Two-way ANOVA
	EBC-1 R-CRIZ + JNJ vs. EBC-1 R-	3	>0,9999	ns	Two-

Appendix Figure S11

		PHA + JNJ(1600nM)				way ANOVA
		EBC-1 R-PHA + CRIZ vs. EBC-1 R-PHA + PHA(1600nM)	3	>0,9999	ns	Two-way ANOVA
		EBC-1 R-PHA + CRIZ vs. EBC-1 R-PHA + JNJ(1600nM)	3	>0,9999	ns	Two-way ANOVA
		EBC-1 R-PHA + PHA vs. EBC-1 R-PHA + JNJ(1600nM)	3	>0,9999	ns	Two-way ANOVA
**1**	B	GTL16 WT+CRIZ vs GTL16 R-CRIZ + CRIZ (25nM)	3	<0.0001	***	Two-way ANOVA
		GTL16 WT+CRIZ vs GTL16 R-CRIZ + PHA (25nM)	3	<0.0001	***	Two-way ANOVA
		GTL16 WT+CRIZ vs GTL16 R-CRIZ + JNJ(25nM)	3	<0.0001	***	Two-way ANOVA
		GTL16 WT + PHA vs. GTL16 R-PHA + CRIZ(25nM)	3	<0.0001	***	Two-way ANOVA
		GTL16 WT + PHA vs. GTL16 R-PHA + PHA(25nM)	3	<0.0001	***	Two-way ANOVA
		GTL16 WT + PHA vs. GTL16 R-PHA + JNJ(25nM)	3	<0.0001	***	Two-way ANOVA
		GTL16 R-CRIZ + CRIZ vs. GTL16 R-CRIZ + PHA(25nM)	3	>0,9999	ns	Two-way ANOVA
		GTL16 R-CRIZ + CRIZ vs. GTL16 R-CRIZ + JNJ(25nM)	3	>0,9999	ns	Two-way ANOVA
		GTL16 R-CRIZ + CRIZ vs. GTL16 R-PHA + CRIZ(25nM)	3	>0,9999	ns	Two-way ANOVA
		GTL16 R-CRIZ + CRIZ vs. GTL16 R-PHA + PHA(25nM)	3	>0,9999	ns	Two-way ANOVA
		GTL16 R-CRIZ + CRIZ vs. GTL16 R-PHA + JNJ(25nM)	3	>0,9999	ns	Two-way ANOVA
		GTL16 R-CRIZ + PHA vs. GTL16 R-CRIZ + JNJ(25nM)	3	>0,9999	ns	Two-way ANOVA
		GTL16 R-CRIZ + PHA vs. GTL16 R-PHA + CRIZ(25nM)	3	>0,9999	ns	Two-way ANOVA
		GTL16 R-CRIZ + PHA vs. GTL16 R-PHA + PHA(25nM)	3	>0,9999	ns	Two-way

Appendix Figure S11

					ANOVA
	GTL16 R-CRIZ + PHA vs. GTL16 R-PHA + JNJ(25nM)	3	>0,9999	ns	Two-way ANOVA
	GTL16 R-CRIZ + JNJ vs. GTL16 R-PHA + CRIZ(25nM)	3	>0,9999	ns	Two-way ANOVA
	GTL16 R-CRIZ + JNJ vs. GTL16 R-PHA + PHA(25nM)	3	>0,9999	ns	Two-way ANOVA
	GTL16 R-CRIZ + JNJ vs. GTL16 R-PHA + JNJ(25nM)	3	>0,9999	ns	Two-way ANOVA
	GTL16 R-PHA + CRIZ vs. GTL16 R-PHA + PHA(25nM)	3	>0,9999	ns	Two-way ANOVA
	GTL16 R-PHA + CRIZ vs. GTL16 R-PHA + JNJ(25nM)	3	>0,9999	ns	Two-way ANOVA
	GTL16 R-PHA + PHA vs. GTL16 R-PHA + JNJ(25nM)	3	>0,9999	ns	Two-way ANOVA
	GTL16 WT+CRIZ vs. GTL16 R-CRIZ + CRIZ (50nM)	3	<0.0001	***	Two-way ANOVA
	GTL16 WT+CRIZ vs. GTL16 R-CRIZ + PHA(50nM)	3	<0.0001	***	Two-way ANOVA
	GTL16 WT+CRIZ vs. GTL16 R-CRIZ + JNJ(50nM)	3	<0.0001	***	Two-way ANOVA
	GTL16 WT + PHA vs. GTL16 R-PHA + CRIZ(50nM)	3	<0.0001	***	Two-way ANOVA
	GTL16 WT + PHA vs. GTL16 R-PHA + PHA(50nM)	3	<0.0001	***	Two-way ANOVA
	GTL16 WT + PHA vs. GTL16 R-PHA + JNJ(50nM)	3	<0.0001	***	Two-way ANOVA
	GTL16 R-CRIZ + CRIZ vs. GTL16 R-CRIZ + PHA(50nM)	3	>0,9999	ns	Two-way ANOVA
	GTL16 R-CRIZ + CRIZ vs. GTL16 R-CRIZ + JNJ(50nM)	3	>0,9999	ns	Two-way ANOVA
	GTL16 R-CRIZ + CRIZ vs. GTL16 R-PHA + CRIZ(50nM)	3	>0,9999	ns	Two-way ANOVA
	GTL16 R-CRIZ + CRIZ vs. GTL16 R-PHA + PHA(50nM)	3	>0,9999	ns	Two-way ANOVA
	GTL16 R-CRIZ + CRIZ vs. GTL16 R-	3	>0,9999	ns	Two-

	PHA + JNJ(50nM)				way ANOVA
	GTL16 R-CRIZ + PHA vs. GTL16 R-CRIZ + JNJ(50nM)	3	>0,9999	ns	Two-way ANOVA
	GTL16 R-CRIZ + PHA vs. GTL16 R-PHA + CRIZ(50nM)	3	>0,9999	ns	Two-way ANOVA
	GTL16 R-CRIZ + PHA vs. GTL16 R-PHA + PHA(50nM)	3	>0,9999	ns	Two-way ANOVA
	GTL16 R-CRIZ + PHA vs. GTL16 R-PHA + JNJ(50nM)	3	>0,9999	ns	Two-way ANOVA
	GTL16 R-CRIZ + JNJ vs. GTL16 R-PHA + CRIZ(50nM)	3	>0,9999	ns	Two-way ANOVA
	GTL16 R-CRIZ + JNJ vs. GTL16 R-PHA + PHA(50nM)	3	>0,9999	ns	Two-way ANOVA
	GTL16 R-CRIZ + JNJ vs. GTL16 R-PHA + JNJ(50nM)	3	>0,9999	ns	Two-way ANOVA
	GTL16 R-PHA + CRIZ vs. GTL16 R-PHA + PHA(50nM)	3	>0,9999	ns	Two-way ANOVA
	GTL16 R-PHA + CRIZ vs. GTL16 R-PHA + JNJ(50nM)	3	>0,9999	ns	Two-way ANOVA
	GTL16 R-PHA + PHA vs. GTL16 R-PHA + JNJ(50nM)	3	>0,9999	ns	Two-way ANOVA
	GTL16 WT+CRIZ vs. GTL16 R-CRIZ + CRIZ (100nM)	3	<0.0001	***	Two-way ANOVA
	GTL16 WT+CRIZ vs. GTL16 R-CRIZ + PHA(100nM)	3	<0.0001	***	Two-way ANOVA
	GTL16 WT+CRIZ vs. GTL16 R-CRIZ + JNJ(100nM)	3	<0.0001	***	Two-way ANOVA
	GTL16 R-CRIZ + CRIZ vs. GTL16 R-CRIZ + PHA(100nM)	3	>0,9999	ns	Two-way ANOVA
	GTL16 R-CRIZ + CRIZ vs. GTL16 R-CRIZ + JNJ(100nM)	3	>0,9999	ns	Two-way ANOVA
	GTL16 R-CRIZ + CRIZ vs. GTL16 R-PHA + CRIZ(100nM)	3	>0,9999	ns	Two-way ANOVA
	GTL16 R-CRIZ + CRIZ vs. GTL16 R-PHA + PHA(100nM)	3	>0,9999	ns	Two-way ANOVA

Appendix Figure S11

	GTL16 R-CRIZ + CRIZ vs. GTL16 R-PHA + JNJ(100nM)	3	>0,9999	ns	Two-way ANOVA
	GTL16 R-CRIZ + PHA vs. GTL16 R-CRIZ + JNJ(100nM)	3	>0,9999	ns	Two-way ANOVA
	GTL16 R-CRIZ + PHA vs. GTL16 R-PHA + CRIZ(100nM)	3	0,2861	ns	Two-way ANOVA
	GTL16 R-CRIZ + PHA vs. GTL16 R-PHA + PHA(100nM)	3	>0,9999	ns	Two-way ANOVA
	GTL16 R-CRIZ + PHA vs. GTL16 R-PHA + JNJ(100nM)	3	>0,9999	ns	Two-way ANOVA
	GTL16 R-CRIZ + JNJ vs. GTL16 R-PHA + CRIZ(100nM)	3	>0,9999	ns	Two-way ANOVA
	GTL16 R-CRIZ + JNJ vs. GTL16 R-PHA + PHA(100nM)	3	>0,9999	ns	Two-way ANOVA
	GTL16 R-CRIZ + JNJ vs. GTL16 R-PHA + JNJ(100nM)	3	>0,9999	ns	Two-way ANOVA
	GTL16 R-PHA + CRIZ vs. GTL16 R-PHA + PHA(100nM)	3	0,2156	ns	Two-way ANOVA
	GTL16 R-PHA + CRIZ vs. GTL16 R-PHA + JNJ(100nM)	3	>0,9999	ns	Two-way ANOVA
	GTL16 R-PHA + PHA vs. GTL16 R-PHA + JNJ(100nM)	3	>0,9999	ns	Two-way ANOVA
	GTL16 WT+CRIZ vs. GTL16 R-CRIZ + CRIZ (200nM)	3	<0.0001	***	Two-way ANOVA
	GTL16 WT+CRIZ vs. GTL16 R-CRIZ + PHA(200nM)	3	<0.0001	***	Two-way ANOVA
	GTL16 WT+CRIZ vs. GTL16 R-CRIZ + JNJ(200nM)	3	<0.0001	***	Two-way ANOVA
	GTL16 WT + PHA vs. GTL16 R-PHA + CRIZ(200nM)	3	<0.0001	***	Two-way ANOVA
	GTL16 WT + PHA vs. GTL16 R-PHA + PHA(200nM)	3	<0.0001	***	Two-way ANOVA
	GTL16 WT + PHA vs. GTL16 R-PHA + JNJ(200nM)	3	<0.0001	***	Two-way ANOVA
	GTL16 R-CRIZ + CRIZ vs. GTL16 R-CRIZ + PHA(200nM)	3	>0,9999	ns	Two-way

Appendix Figure S11

					ANOVA
	GTL16 R-CRIZ + CRIZ vs. GTL16 R-CRIZ + JNJ(200nM)	3	>0,9999	ns	Two-way ANOVA
	GTL16 R-CRIZ + CRIZ vs. GTL16 R-PHA + CRIZ(200nM)	3	0,0883	ns	Two-way ANOVA
	GTL16 R-CRIZ + CRIZ vs. GTL16 R-PHA + PHA(200nM)	3	>0,9999	ns	Two-way ANOVA
	GTL16 R-CRIZ + CRIZ vs. GTL16 R-PHA + JNJ(200nM)	3	>0,9999	ns	Two-way ANOVA
	GTL16 R-CRIZ + PHA vs. GTL16 R-CRIZ + JNJ(200nM)	3	>0,9999	ns	Two-way ANOVA
	GTL16 R-CRIZ + PHA vs. GTL16 R-PHA + CRIZ(200nM)	3	0,0016	**	Two-way ANOVA
	GTL16 R-CRIZ + PHA vs. GTL16 R-PHA + PHA(200nM)	3	0,1198	ns	Two-way ANOVA
	GTL16 R-CRIZ + PHA vs. GTL16 R-PHA + JNJ(200nM)	3	0,0883	ns	Two-way ANOVA
	GTL16 R-CRIZ + JNJ vs. GTL16 R-PHA + CRIZ(200nM)	3	0,0016	**	Two-way ANOVA
	GTL16 R-CRIZ + JNJ vs. GTL16 R-PHA + PHA(200nM)	3	0,1198	ns	Two-way ANOVA
	GTL16 R-CRIZ + JNJ vs. GTL16 R-PHA + JNJ(200nM)	3	0,0883	ns	Two-way ANOVA
	GTL16 R-PHA + CRIZ vs. GTL16 R-PHA + PHA(200nM)	3	>0,9999	ns	Two-way ANOVA
	GTL16 R-PHA + CRIZ vs. GTL16 R-PHA + JNJ(200nM)	3	>0,9999	ns	Two-way ANOVA
	GTL16 R-PHA + PHA vs. GTL16 R-PHA + JNJ(200nM)	3	>0,9999	ns	Two-way ANOVA
	GTL16 WT+CRIZ vs. GTL16 R-CRIZ + CRIZ(400nM)	3	<0.0001	***	Two-way ANOVA
	GTL16 WT+CRIZ vs. GTL16 R-CRIZ + PHA(400nM)	3	<0.0001	***	Two-way ANOVA
	GTL16 WT+CRIZ vs. GTL16 R-CRIZ + JNJ(400nM)	3	<0.0001	***	Two-way ANOVA
	GTL16 WT + PHA vs. GTL16 R-	3	0,0002	***	Two-

Appendix Figure S11

	PHA + CRIZ(400nM)				way ANOVA
	GTL16 WT + PHA vs. GTL16 R-PHA + PHA(400nM)	3	<0.0001	***	Two-way ANOVA
	GTL16 WT + PHA vs. GTL16 R-PHA + JNJ(400nM)	3	<0.0001	***	Two-way ANOVA
	GTL16 R-CRIZ + CRIZ vs. GTL16 R-CRIZ + PHA(400nM)	3	>0,9999	ns	Two-way ANOVA
	GTL16 R-CRIZ + CRIZ vs. GTL16 R-CRIZ + JNJ(400nM)	3	>0,9999	ns	Two-way ANOVA
	GTL16 R-CRIZ + CRIZ vs. GTL16 R-PHA + CRIZ(400nM)	3	0,0009	***	Two-way ANOVA
	GTL16 R-CRIZ + CRIZ vs. GTL16 R-PHA + PHA(400nM)	3	0,1029	ns	Two-way ANOVA
	GTL16 R-CRIZ + CRIZ vs. GTL16 R-PHA + JNJ(400nM)	3	0,0646	ns	Two-way ANOVA
	GTL16 R-CRIZ + PHA vs. GTL16 R-CRIZ + JNJ(400nM)	3	>0,9999	ns	Two-way ANOVA
	GTL16 R-CRIZ + PHA vs. GTL16 R-PHA + CRIZ(400nM)	3	<0,0001	***	Two-way ANOVA
	GTL16 R-CRIZ + PHA vs. GTL16 R-PHA + PHA(400nM)	3	0,0002	***	Two-way ANOVA
	GTL16 R-CRIZ + PHA vs. GTL16 R-PHA + JNJ(400nM)	3	<0,0001	***	Two-way ANOVA
	GTL16 R-CRIZ + JNJ vs. GTL16 R-PHA + CRIZ(400nM)	3	<0,0001	***	Two-way ANOVA
	GTL16 R-CRIZ + JNJ vs. GTL16 R-PHA + PHA(400nM)	3	0,0001	***	Two-way ANOVA
	GTL16 R-CRIZ + JNJ vs. GTL16 R-PHA + JNJ(400nM)	3	<0,0001	***	Two-way ANOVA
	GTL16 R-PHA + CRIZ vs. GTL16 R-PHA + PHA(400nM)	3	>0,9999	ns	Two-way ANOVA
	GTL16 R-PHA + CRIZ vs. GTL16 R-PHA + JNJ(400nM)	3	>0,9999	ns	Two-way ANOVA
	GTL16 R-PHA + PHA vs. GTL16 R-PHA + JNJ(400nM)	3	>0,9999	ns	Two-way ANOVA

Appendix Figure S11

	GTL16 WT+CRIZ vs. GTL16 R-CRIZ + CRIZ(800nM)	3	<0.0001	***	Two-way ANOVA
	GTL16 WT+CRIZ vs. GTL16 R-CRIZ + PHA(800nM)	3	<0.0001	***	Two-way ANOVA
	GTL16 WT+CRIZ vs. GTL16 R-CRIZ + JNJ(800nM)	3	<0.0001	***	Two-way ANOVA
	GTL16 WT + PHA vs. GTL16 R-PHA + CRIZ(800nM)	3	0,0552	ns	Two-way ANOVA
	GTL16 WT + PHA vs. GTL16 R-PHA + PHA(800nM)	3	<0.0001	***	Two-way ANOVA
	GTL16 WT + PHA vs. GTL16 R-PHA + JNJ(800nM)	3	<0.0001	***	Two-way ANOVA
	GTL16 R-CRIZ + CRIZ vs. GTL16 R-CRIZ + PHA(800nM)	3	0,0339	*	Two-way ANOVA
	GTL16 R-CRIZ + CRIZ vs. GTL16 R-CRIZ + JNJ(800nM)	3	0,0013	**	Two-way ANOVA
	GTL16 R-CRIZ + CRIZ vs. GTL16 R-PHA + CRIZ(800nM)	3	0,0042	**	Two-way ANOVA
	GTL16 R-CRIZ + CRIZ vs. GTL16 R-PHA + PHA(800nM)	3	>0,9999	ns	Two-way ANOVA
	GTL16 R-CRIZ + CRIZ vs. GTL16 R-PHA + JNJ(800nM)	3	>0,9999	ns	Two-way ANOVA
	GTL16 R-CRIZ + PHA vs. GTL16 R-CRIZ + JNJ(800nM)	3	>0,9999	ns	Two-way ANOVA
	GTL16 R-CRIZ + PHA vs. GTL16 R-PHA + CRIZ(800nM)	3	<0,0001	***	Two-way ANOVA
	GTL16 R-CRIZ + PHA vs. GTL16 R-PHA + PHA(800nM)	3	<0,0001	***	Two-way ANOVA
	GTL16 R-CRIZ + PHA vs. GTL16 R-PHA + JNJ(800nM)	3	<0,0001	***	Two-way ANOVA
	GTL16 R-CRIZ + JNJ vs. GTL16 R-PHA + CRIZ(800nM)	3	<0,0001	***	Two-way ANOVA
	GTL16 R-CRIZ + JNJ vs. GTL16 R-PHA + PHA(800nM)	3	<0,0001	***	Two-way ANOVA
	GTL16 R-CRIZ + JNJ vs. GTL16 R-PHA + JNJ(800nM)	3	<0,0001	***	Two-way

Appendix Figure S11

					ANOVA
	GTL16 R-PHA + CRIZ vs. GTL16 R-PHA + PHA(800nM)	3	>0,9999	ns	Two-way ANOVA
	GTL16 R-PHA + CRIZ vs. GTL16 R-PHA + JNJ(800nM)	3	0,6391	ns	Two-way ANOVA
	GTL16 R-PHA + PHA vs. GTL16 R-PHA + JNJ(800nM)	3	>0,9999	ns	Two-way ANOVA
	GTL16 WT+CRIZ vs. GTL16 R-CRIZ + CRIZ(1600nM)	3	0.0001	***	Two-way ANOVA
	GTL16 WT+CRIZ vs. GTL16 R-CRIZ + PHA(1600nM)	3	<0.0001	***	Two-way ANOVA
	GTL16 WT+CRIZ vs. GTL16 R-CRIZ + JNJ(1600nM)	3	<0.0001	***	Two-way ANOVA
	GTL16 WT + PHA vs. GTL16 R-PHA + CRIZ(1600nM)	3	>0,9999	ns	Two-way ANOVA
	GTL16 WT + PHA vs. GTL16 R-PHA + PHA(1600nM)	3	0,0005	***	Two-way ANOVA
	GTL16 WT + PHA vs. GTL16 R-PHA + JNJ(1600nM)	3	0,0001	***	Two-way ANOVA
	GTL16 R-CRIZ + CRIZ vs. GTL16 R-CRIZ + PHA(1600nM)	3	<0,0001	***	Two-way ANOVA
	GTL16 R-CRIZ + CRIZ vs. GTL16 R-CRIZ + JNJ(1600nM)	3	<0,0001	***	Two-way ANOVA
	GTL16 R-CRIZ + CRIZ vs. GTL16 R-PHA + CRIZ(1600nM)	3	0,9314	ns	Two-way ANOVA
	GTL16 R-CRIZ + CRIZ vs. GTL16 R-PHA + PHA(1600nM)	3	>0,9999	ns	Two-way ANOVA
	GTL16 R-CRIZ + CRIZ vs. GTL16 R-PHA + JNJ(1600nM)	3	>0,9999	ns	Two-way ANOVA
	GTL16 R-CRIZ + PHA vs. GTL16 R-CRIZ + JNJ(1600nM)	3	>0,9999	ns	Two-way ANOVA
	GTL16 R-CRIZ + PHA vs. GTL16 R-PHA + CRIZ(1600nM)	3	<0,0001	***	Two-way ANOVA
	GTL16 R-CRIZ + PHA vs. GTL16 R-PHA + PHA(1600nM)	3	<0,0001	***	Two-way ANOVA
	GTL16 R-CRIZ + PHA vs. GTL16 R-	3	<0,0001	***	Two-

Appendix Figure S11

		PHA + JNJ(1600nM)				way ANOVA
		GTL16 R-CRIZ + JNJ vs. GTL16 R-PHA + CRIZ(1600nM)	3	<0,0001	***	Two-way ANOVA
		GTL16 R-CRIZ + JNJ vs. GTL16 R-PHA + PHA(1600nM)	3	<0,0001	***	Two-way ANOVA
		GTL16 R-CRIZ + JNJ vs. GTL16 R-PHA + JNJ(1600nM)	3	<0,0001	***	Two-way ANOVA
		GTL16 R-PHA + CRIZ vs. GTL16 R-PHA + PHA(1600nM)	3	0,4312	ns	Two-way ANOVA
		GTL16 R-PHA + CRIZ vs. GTL16 R-PHA + JNJ(1600nM)	3	0,1613	ns	Two-way ANOVA
		GTL16 R-PHA + PHA vs. GTL16 R-PHA + JNJ(1600nM)	3	>0,9999	ns	Two-way ANOVA
**1**	C	SG16 WT + CRIZ vs. SG16 WT + PHA (25nM)	3	0,3403	ns	Two-way ANOVA
		SG16 WT + CRIZ vs. SG16 WT + JNJ(25nM)	3	>0,9999	ns	Two-way ANOVA
		SG16 WT + CRIZ vs. SG16 R-JNJ + CRIZ(25nM)	3	<0,0001	***	Two-way ANOVA
		SG16 WT + CRIZ vs. SG16 R-JNJ + PHA(25nM)	3	<0,0001	***	Two-way ANOVA
		SG16 WT + CRIZ vs. SG16 R-JNJ + JNJ(25nM)	3	<0,0001	***	Two-way ANOVA
		SG16 WT + PHA vs. SG16 WT + JNJ(25nM)	3	>0,9999	ns	Two-way ANOVA
		SG16 WT + PHA vs. SG16 R-JNJ + CRIZ(25nM)	3	<0,0001	***	Two-way ANOVA
		SG16 WT + PHA vs. SG16 R-JNJ + PHA(25nM)	3	<0,0001	***	Two-way ANOVA
		SG16 WT + PHA vs. SG16 R-JNJ + JNJ(25nM)	3	<0,0001	***	Two-way ANOVA
		SG16 WT + JNJ vs. SG16 R-JNJ + CRIZ(25nM)	3	<0,0001	***	Two-way ANOVA
		SG16 WT + JNJ vs. SG16 R-JNJ + PHA(25nM)	3	<0,0001	***	Two-way

Appendix Figure S11

					ANOVA
	SG16 WT + JNJ vs. SG16 R-JNJ + JNJ(25nM)	3	<0,0001	***	Two-way ANOVA
	SG16 R-JNJ + CRIZ vs. SG16 R-JNJ + PHA(25nM)	3	>0,9999	ns	Two-way ANOVA
	SG16 R-JNJ + CRIZ vs. SG16 R-JNJ + JNJ(25nM)	3	>0,9999	ns	Two-way ANOVA
	SG16 R-JNJ + PHA vs. SG16 R-JNJ + JNJ(25nM)	3	>0,9999	ns	Two-way ANOVA
	SG16 WT + CRIZ vs. SG16 WT + PHA (50nM)	3	>0,9999	ns	Two-way ANOVA
	SG16 WT + CRIZ vs. SG16 WT + JNJ(50nM)	3	>0,9999	ns	Two-way ANOVA
	SG16 WT + CRIZ vs. SG16 R-JNJ + CRIZ(50nM)	3	<0,0001	***	Two-way ANOVA
	SG16 WT + CRIZ vs. SG16 R-JNJ + PHA(50nM)	3	<0,0001	***	Two-way ANOVA
	SG16 WT + CRIZ vs. SG16 R-JNJ + JNJ(50nM)	3	<0,0001	***	Two-way ANOVA
	SG16 WT + PHA vs. SG16 WT + JNJ(50nM)	3	>0,9999	ns	Two-way ANOVA
	SG16 WT + PHA vs. SG16 R-JNJ + CRIZ(50nM)	3	<0,0001	***	
	SG16 WT + PHA vs. SG16 R-JNJ + PHA(50nM)	3	<0,0001	***	Two-way ANOVA
	SG16 WT + PHA vs. SG16 R-JNJ + JNJ(50nM)	3	<0,0001	***	Two-way ANOVA
	SG16 WT + JNJ vs. SG16 R-JNJ + CRIZ(50nM)	3	<0,0001	***	Two-way ANOVA
	SG16 WT + JNJ vs. SG16 R-JNJ + PHA(50nM)	3	<0,0001	***	Two-way ANOVA
	SG16 WT + JNJ vs. SG16 R-JNJ + JNJ(50nM)	3	<0,0001	***	Two-way ANOVA
	SG16 R-JNJ + CRIZ vs. SG16 R-JNJ + PHA(50nM)	3	>0,9999	ns	Two-way ANOVA
	SG16 R-JNJ + CRIZ vs. SG16 R-JNJ + JNJ(50nM)	3	>0,9999	ns	Two-way

Appendix Figure S11

					ANOVA
	SG16 R-JNJ + PHA vs. SG16 R-JNJ + JNJ(50nM)	3	>0,9999	ns	Two-way ANOVA
	SG16 WT + CRIZ vs. SG16 WT + PHA (100nM)	3	>0,9999	ns	Two-way ANOVA
	SG16 WT + CRIZ vs. SG16 WT + JNJ(100nM)	3	>0,9999	ns	Two-way ANOVA
	SG16 WT + CRIZ vs. SG16 R-JNJ + CRIZ(100nM)	3	<0,0001	***	Two-way ANOVA
	SG16 WT + CRIZ vs. SG16 R-JNJ + PHA(100nM)	3	<0,0001	***	Two-way ANOVA
	SG16 WT + CRIZ vs. SG16 R-JNJ + JNJ(100nM)	3	<0,0001	***	Two-way ANOVA
	SG16 WT + PHA vs. SG16 WT + JNJ(100nM)	3	>0,9999	ns	Two-way ANOVA
	SG16 WT + PHA vs. SG16 R-JNJ + CRIZ(100nM)	3	<0,0001	***	Two-way ANOVA
	SG16 WT + PHA vs. SG16 R-JNJ + PHA(100nM)	3	<0,0001	***	Two-way ANOVA
	SG16 WT + PHA vs. SG16 R-JNJ + JNJ(100nM)	3	<0,0001	***	Two-way ANOVA
	SG16 WT + JNJ vs. SG16 R-JNJ + CRIZ(100nM)	3	<0,0001	***	Two-way ANOVA
	SG16 WT + JNJ vs. SG16 R-JNJ + PHA(100nM)	3	<0,0001	***	Two-way ANOVA
	SG16 WT + JNJ vs. SG16 R-JNJ + JNJ(100nM)	3	<0,0001	***	Two-way ANOVA
	SG16 R-JNJ + CRIZ vs. SG16 R-JNJ + PHA(100nM)	3	>0,9999	ns	Two-way ANOVA
	SG16 R-JNJ + CRIZ vs. SG16 R-JNJ + JNJ(100nM)	3	>0,9999	ns	
	SG16 R-JNJ + PHA vs. SG16 R-JNJ + JNJ(100nM)	3	>0,9999	ns	Two-way ANOVA
	SG16 WT + CRIZ vs. SG16 WT + PHA (200nM)	3	>0,9999	ns	Two-way ANOVA
	SG16 WT + CRIZ vs. SG16 WT + JNJ(200nM)	3	>0,9999	ns	Two-way

Appendix Figure S11

					ANOVA
	SG16 WT + CRIZ vs. SG16 R-JNJ + CRIZ(200nM)	3	<0,0001	***	Two-way ANOVA
	SG16 WT + CRIZ vs. SG16 R-JNJ + PHA(200nM)	3	<0,0001	***	Two-way ANOVA
	SG16 WT + CRIZ vs. SG16 R-JNJ + JNJ(200nM)	3	<0,0001	***	Two-way ANOVA
	SG16 WT + PHA vs. SG16 WT + JNJ(200nM)	3	>0,9999	ns	Two-way ANOVA
	SG16 WT + PHA vs. SG16 R-JNJ + CRIZ(200nM)	3	<0,0001	***	Two-way ANOVA
	SG16 WT + PHA vs. SG16 R-JNJ + PHA(200nM)	3	<0,0001	***	Two-way ANOVA
	SG16 WT + PHA vs. SG16 R-JNJ + JNJ(200nM)	3	<0,0001	***	Two-way ANOVA
	SG16 WT + JNJ vs. SG16 R-JNJ + CRIZ(200nM)	3	<0,0001	***	Two-way ANOVA
	SG16 WT + JNJ vs. SG16 R-JNJ + PHA(200nM)	3	<0,0001	***	Two-way ANOVA
	SG16 WT + JNJ vs. SG16 R-JNJ + JNJ(200nM)	3	<0,0001	***	Two-way ANOVA
	SG16 R-JNJ + CRIZ vs. SG16 R-JNJ + PHA(200nM)	3	>0,9999	ns	Two-way ANOVA
	SG16 R-JNJ + CRIZ vs. SG16 R-JNJ + JNJ(200nM)	3	>0,9999	ns	Two-way ANOVA
	SG16 R-JNJ + PHA vs. SG16 R-JNJ + JNJ(200nM)	3	>0,9999	ns	Two-way ANOVA
	SG16 WT + CRIZ vs. SG16 WT + PHA (400nM)	3	>0,9999	ns	Two-way ANOVA
	SG16 WT + CRIZ vs. SG16 WT + JNJ(400nM)	3	>0,9999	ns	Two-way ANOVA
	SG16 WT + CRIZ vs. SG16 R-JNJ + CRIZ(400nM)	3	<0,0001	***	Two-way ANOVA
	SG16 WT + CRIZ vs. SG16 R-JNJ + PHA(400nM)	3	<0,0001	***	Two-way ANOVA
	SG16 WT + CRIZ vs. SG16 R-JNJ +	3	<0,0001	***	Two-

Appendix Figure S11

	JNJ(400nM)				way ANOVA
	SG16 WT + PHA vs. SG16 WT + JNJ(400nM)	3	>0,9999	ns	Two-way ANOVA
	SG16 WT + PHA vs. SG16 R-JNJ + CRIZ(400nM)	3	<0,0001	***	Two-way ANOVA
	SG16 WT + PHA vs. SG16 R-JNJ + PHA(400nM)	3	<0,0001	***	Two-way ANOVA
	SG16 WT + PHA vs. SG16 R-JNJ + JNJ(400nM)	3	<0,0001	***	Two-way ANOVA
	SG16 WT + JNJ vs. SG16 R-JNJ + CRIZ(400nM)	3	<0,0001	***	Two-way ANOVA
	SG16 WT + JNJ vs. SG16 R-JNJ + PHA(400nM)	3	<0,0001	***	Two-way ANOVA
	SG16 WT + JNJ vs. SG16 R-JNJ + JNJ(400nM)	3	<0,0001	***	Two-way ANOVA
	SG16 R-JNJ + CRIZ vs. SG16 R-JNJ + PHA(400nM)	3	>0,9999	ns	Two-way ANOVA
	SG16 R-JNJ + CRIZ vs. SG16 R-JNJ + JNJ(400nM)	3	>0,9999	ns	Two-way ANOVA
	SG16 R-JNJ + PHA vs. SG16 R-JNJ + JNJ(400nM)	3	>0,9999	ns	Two-way ANOVA
	SG16 WT + CRIZ vs. SG16 WT + PHA (800nM)	3	>0,9999	ns	Two-way ANOVA
	SG16 WT + CRIZ vs. SG16 WT + JNJ(800nM)	3	>0,9999	ns	Two-way ANOVA
	SG16 WT + CRIZ vs. SG16 R-JNJ + CRIZ(800nM)	3	<0,0001	***	Two-way ANOVA
	SG16 WT + CRIZ vs. SG16 R-JNJ + PHA(800nM)	3	<0,0001	***	Two-way ANOVA
	SG16 WT + CRIZ vs. SG16 R-JNJ + JNJ(800nM)	3	<0,0001	***	Two-way ANOVA
	SG16 WT + PHA vs. SG16 WT + JNJ(800nM)	3	>0,9999	ns	Two-way ANOVA
	SG16 WT + PHA vs. SG16 R-JNJ + CRIZ(800nM)	3	<0,0001	***	Two-way ANOVA

Appendix Figure S11

	SG16 WT + PHA vs. SG16 R-JNJ + PHA(800nM)	3	<0,0001	***	Two-way ANOVA
	SG16 WT + PHA vs. SG16 R-JNJ + JNJ(800nM)	3	<0,0001	***	Two-way ANOVA
	SG16 WT + JNJ vs. SG16 R-JNJ + CRIZ(800nM)	3	<0,0001	***	Two-way ANOVA
	SG16 WT + JNJ vs. SG16 R-JNJ + PHA(800nM)	3	<0,0001	***	Two-way ANOVA
	SG16 WT + JNJ vs. SG16 R-JNJ + JNJ(800nM)	3	<0,0001	***	Two-way ANOVA
	SG16 R-JNJ + CRIZ vs. SG16 R-JNJ + PHA(800nM)	3	<0,0001	***	Two-way ANOVA
	SG16 R-JNJ + CRIZ vs. SG16 R-JNJ + JNJ(800nM)	3	<0,0001	***	Two-way ANOVA
	SG16 R-JNJ + PHA vs. SG16 R-JNJ + JNJ(800nM)	3	>0,9999	ns	Two-way ANOVA
	SG16 WT + CRIZ vs. SG16 WT + PHA (1600nM)	3	0,0015	**	Two-way ANOVA
	SG16 WT + CRIZ vs. SG16 WT + JNJ(1600nM)	3	0,0008	***	Two-way ANOVA
	SG16 WT + CRIZ vs. SG16 R-JNJ + CRIZ(1600nM)	3	0,439	ns	Two-way ANOVA
	SG16 WT + CRIZ vs. SG16 R-JNJ + PHA(1600nM)	3	<0,0001	***	Two-way ANOVA
	SG16 WT + CRIZ vs. SG16 R-JNJ + JNJ(1600nM)	3	<0,0001	***	Two-way ANOVA
	SG16 WT + PHA vs. SG16 WT + JNJ(1600nM)	3	>0,9999	ns	Two-way ANOVA
	SG16 WT + PHA vs. SG16 R-JNJ + CRIZ(1600nM)	3	>0,9999	ns	Two-way ANOVA
	SG16 WT + PHA vs. SG16 R-JNJ + PHA(1600nM)	3	<0,0001	***	Two-way ANOVA
	SG16 WT + PHA vs. SG16 R-JNJ + JNJ(1600nM)	3	<0,0001	***	Two-way ANOVA
	SG16 WT + JNJ vs. SG16 R-JNJ + CRIZ(1600nM)	3	0,7132	ns	Two-way

Appendix Figure S11

						ANOVA
		SG16 WT + JNJ vs. SG16 R-JNJ + PHA(1600nM)	3	<0,0001	***	Two-way ANOVA
		SG16 WT + JNJ vs. SG16 R-JNJ + JNJ(1600nM)	3	<0,0001	***	Two-way ANOVA
		SG16 R-JNJ + CRIZ vs. SG16 R-JNJ + PHA(1600nM)	3	<0,0001	***	Two-way ANOVA
		SG16 R-JNJ + CRIZ vs. SG16 R-JNJ + JNJ(1600nM)	3	<0,0001	***	Two-way ANOVA
		SG16 R-JNJ + PHA vs. SG16 R-JNJ + JNJ(1600nM)	3	>0,9999	ns	Two-way ANOVA
**1**	D	SG16 WT vs. SG16 WT 5-AZA	3	<0,0001	***	One-way ANOVA
		SG16 WT vs. SG16 R-JNJ	3	<0,0001	***	One-way ANOVA
		EBC-1 WT vs. EBC-1 WT 5-AZA	3	<0,0001	***	One-way ANOVA
		EBC-1 WT vs. EBC-1 R-CRIZ	3	<0,0001	***	One-way ANOVA
		EBC-1 WT vs. EBC-1 R-PHA	3	<0,0001	***	One-way ANOVA
**1**	E	SG16 WT vs. SG16 WT 5-AZA	3	0,000014	***	T-TEST
		EBC-1 WT vs. EBC-1 WT 5-AZA	3	0,0009	***	T-TEST
**2**	A	R-CRIZ (anti-miR vs ctrl)	4	0,0028	**	T-TEST
	A	R-PHA (anti-miR vs ctrl)	4	0,0019	**	T-TEST
**2**	B	R-CRIZ (anti-miR vs ctrl)	4	0,0135	*	T-TEST
	B	R-PHA (anti-miR vs ctrl)	4	0,0082	**	T-TEST
**2**	C	R-JNJ (anti-miR vs ctrl)	4	0,0056	**	T-TEST
**2**	D	miR-205 vs ctrl (PHA)	4	0,0008	***	T-TEST
	D	miR-205 vs ctrl (CRIZ)	4	0,0000005	***	T-TEST
**2**	E	miR-205 vs ctrl (PHA)	4	0,000004	***	T-TEST
	E	miR-205 vs ctrl (CRIZ)	4	0,005	**	T-TEST
**2**	F	miR-205 vs ctrl (JNJ)	4	0,0006	***	T-TEST
**2**	G	RCRIZ pCDH + CRIZ vs. RCRIZ pCDH-anti-miR-205 + CRIZ (DAY12)	6	<0,0001	***	Two-way ANOVA
		RCRIZ pCDH + CRIZ vs. RCRIZ pCDH-anti-miR-205 + CRIZ (DAY18)	6	<0,0001	***	Two-way ANOVA
**2**	H	pCDH NT vs. pCDH CRIZ (DAY19)	6	<0,0001	***	Two-

Appendix Figure S11

						way ANOVA
		pCDH NT vs. pCDH miR-205 NT(DAY19)	6	>0,9999	ns	Two-way ANOVA
		pCDH NT vs. pCDH miR-205 CRIZ(DAY19)	6	0,7628	ns	Two-way ANOVA
		pCDH CRIZ vs. pCDH miR-205 NT(DAY19)	6	<0,0001	***	Two-way ANOVA
		pCDH CRIZ vs. pCDH miR-205 CRIZ(DAY19)	6	0,0006	***	Two-way ANOVA
		pCDH miR-205 NT vs. pCDH miR-205 CRIZ(DAY19)	6	>0,9999	ns	Two-way ANOVA
		pCDH NT vs. pCDH CRIZ (DAY26)	6	<0,0001	***	Two-way ANOVA
		pCDH NT vs. pCDH miR-205 NT(DAY26)	6	<0,0001	***	Two-way ANOVA
		pCDH NT vs. pCDH miR-205 CRIZ(DAY26)	6	0,0052	**	Two-way ANOVA
		pCDH CRIZ vs. pCDH miR-205 NT(DAY26)	6	<0,0001	***	Two-way ANOVA
		pCDH CRIZ vs. pCDH miR-205 CRIZ(DAY26)	6	<0,0001	***	Two-way ANOVA
		pCDH miR-205 NT vs. pCDH miR-205 CRIZ(DAY26)	6	0,0682	ns	Two-way ANOVA
**3**	A	pCDH ERRFI1 vs pCDH (R-CRIZ)	4	0,00003	***	T-TEST
	A	pCDH ERRFI1 vs pCDH (R-PHA)	4	0,0008	***	T-TEST
	A	CRIZ vs NT (WT)	4	0,0010	***	T-TEST
	A	PHA vs NT (WT)	4	0,0009	***	T-TEST
**3**	B	pCDH ERRFI1 vs pCDH (R-JNJ)	4	<0,00001	***	T-TEST
	B	JNJ vs NT (WT)	4	4,69773E-07	***	T-TEST
**3**	C	siERRFI1 vs siCtrl (EBC-1)	4	0,0045	**	T-TEST
	C	siERRFI1 vs siCtrl (EBC-1)	4	0,000033	***	T-TEST
**3**	E	miR-205 vs Ctrl	6	2,44483E-05	***	T-TEST
		miR-200c vs Ctrl	3	0.001	***	T-TEST
**3**	F	miR-205 vs Ctrl (wt ERRFI1 3’UTR)	6	2,44483E-05	***	T-TEST
		miR-205 vs Ctrl (delta ERRFI1 3’UTR)	3	0,101088202	ns	T-TEST

Appendix Figure S11

		miR-205 vs Ctrl (mut ERRFI1 3'UTR)	3	0,271964743	ns	T-TEST
**3**	H	WT vs. RCRIZ(1.56nM)	4	>0,9999	ns	Two-way ANOVA
		WT vs. RPHA(1.56nM)	4	0,1206	ns	Two-way ANOVA
		WT vs. RCRIZ(3.125nM)	4	0,1389	ns	Two-way ANOVA
		WT vs. RPHA(3.125nM)	4	0,012	*	Two-way ANOVA
		WT vs. RCRIZ(6.25nM)	4	0,0007	***	Two-way ANOVA
		WT vs. RPHA(6.25nM)	4	<0,0001	***	Two-way ANOVA
		WT vs. RCRIZ(12.5nM)	4	<0,0001	***	Two-way ANOVA
		WT vs. RPHA(12.5nM)	4	<0,0001	***	Two-way ANOVA
		WT vs. RCRIZ(25nM)	4	<0,0001	***	Two-way ANOVA
		WT vs. RPHA(25nM)	4	<0,0001	***	Two-way ANOVA
		WT vs. RCRIZ (50nM)	4	<0,0001	***	Two-way ANOVA
		WT vs. RPHA(50nM)	4	<0,0001	***	Two-way ANOVA
		WT vs. RCRIZ (100nM)	4	<0,0001	***	Two-way ANOVA
		WT vs. RPHA (100nM)	4	<0,0001	***	Two-way ANOVA
**3**	I	WT vs. RJNJ (1,56nM)	4	0,3348	ns	Two-way ANOVA
		WT vs. RJNJ(3,12nM)	4	0,0015	**	Two-way ANOVA
		WT vs. RJNJ(6,25nM)	4	<0,0001	***	Two-way ANOVA

Appendix Figure S11

		WT vs. RJNJ(12,5nM)	4	<0,0001	***	Two-way ANOVA
		WT vs. RJNJ(25nM)	4	<0,0001	***	Two-way ANOVA
		WT vs. RJNJ(50nM)	4	<0,0001	***	Two-way ANOVA
		WT vs. RJNJ(100nM)	4	0,001	***	Two-way ANOVA
**4**	B		3	0,003	**	T-TEST

T-TEST was performed with Microsoft Excel Software; One-way and Two-way ANOVA (followed by Bonferroni ‘s multiple comparison test) were performed with GraphPad software.

Appendix Figure S12

Figure	Panel	experiment	n-value	p-value	Significativity	TEST
**EV1**	B	R-CRIZ vs WT	3	0,00000009	***	T-TEST
	B	R-PHA vs WT	3	0,00001	***	T-TEST
	C	R-CRIZ vs WT	3	0,0002	***	T-TEST
	C	R-PHA vs WT	3	0,000037	***	T-TEST
	D	R-JNJ vs WT	3	1,7123E-05	***	T-TEST
	E	R-PHA vs WT	3	0,000001	***	T-TEST
	F	R-CRIZ vs WT	3	1,3026E-07	***	T-TEST
**EV2**	B	R-PHA vs WT	3	0,000514	***	T-TEST
	C	R-CRIZ vs WT	3	0,000137	***	T-TEST
**EV3**	A	pCDH-ERRFI1 vs pCDH	3	0,010103	*	T-TEST
	B	pCDH vs pCDH-ERRFI1 (DAY12)	6	0,0004	***	Two-way ANOVA
	B	pCDH vs pCDH-ERRFI1 (DAY18)	6	<0,0001	***	Two-way ANOVA

T-TEST was performed with Microsoft Excel Software; One-way and Two-way ANOVA (followed by Bonferroni ‘s multiple comparison test) were performed with GraphPad software.

Appendix Figure S13

Figure	Panel	experiment	n-value	p-value	Significativity	TEST
**S1**	A	WT vs. R-PHA 1,95 nM	4	<0,0001	***	Two-way ANOVA
		WT vs. R-PHA 3,9 nM	4	<0,0001	***	Two-way ANOVA
		WT vs. R-PHA 7,8 nM	4	<0,0001	***	Two-way ANOVA
		WT vs. R-PHA 15,6 nM	4	<0,0001	***	Two-way ANOVA
		WT vs. R-PHA 31 nM	4	<0,0001	***	Two-way ANOVA
		WT vs. R-PHA 62,5 nM	4	<0,0001	***	Two-way ANOVA
		WT vs. R-PHA 125 nM	4	0,0099	**	Two-way ANOVA
		WT vs. R-PHA 250 nM	4	>0,9999	ns	Two-way ANOVA
		WT vs. R-PHA 500 nM	4	>0,9999	ns	Two-way ANOVA
		WT vs. R-PHA 1000 nM	4	>0,9999	ns	Two-way ANOVA
**S1**	B	WT vs. R-CRIZ 0.195 nM	4	>0,9999	ns	Two-way ANOVA
		WT vs. R-CRIZ 0.39 nM	4	0,147	ns	Two-way ANOVA
		WT vs. R-CRIZ 0.78 nM	4	0,0076	**	Two-way ANOVA
		WT vs. R-CRIZ 1.56 nM	4	0,147	ns	Two-way ANOVA
		WT vs. R-CRIZ 3.1 nM	4	<0,0001	***	Two-way ANOVA
		WT vs. R-CRIZ 6.25 nM	4	<0,0001	***	Two-way ANOVA
		WT vs. R-CRIZ 12.5 nM	4	<0,0001	***	Two-way ANOVA
		WT vs. R-CRIZ 25 nM	4	<0,0001	***	Two-way ANOVA
		WT vs. R-CRIZ 50 nM	4	<0,0001	***	Two-way ANOVA
		WT vs. R-CRIZ 100 nM	4	0,0041	**	Two-way ANOVA
**S3**	A	Anti-miR-205 vs Ctrl (R-CRIZ)	3	0,00003	***	T-TEST
	A	Anti-miR-205 vs Ctrl (R-PHA)	3	0,0001	***	T-TEST
	B	Anti-miR-205 vs Ctrl (R-CRIZ)	3	0,0009	***	T-TEST
	B	Anti-miR-205 vs Ctrl (R-PHA)	3	0,0001	***	T-TEST
	C	Anti-miR-205 vs Ctrl (R-JNJ)	3	7,99E-05	***	T-TEST
	D	miR-205 vs Ctrl	3	5,94261E-05	***	T-TEST
	E	miR-205 vs Ctrl	3	1,15821E-05	***	T-TEST

Appendix Figure S13

	F	miR-205 vs ctrl	3	1,2073E-07		T-TEST
**S4**	A	pCDH vs pCDH-ANTI-miR-205	3	0,000162	***	T-TEST
	B	pCDH vs pCDH-miR-205	3	1,46858E-09	***	T-TEST
**S7**	B	pCDH vs pCDH-ERRFI1 (R-CRIZ)	4	0,02	*	T-TEST
		pCDH vs pCDH-ERRFI1 (R-PHA)	4	0.051	ns	T-TEST
**S8**	A	miR-205 vs Ctrl	3	<0.0001	***	T-TEST
**S9**		WT vs. R-CRIZ (1,56nM)	4	0,0148	*	Two-way ANOVA
		WT vs. R-CRIZ(3,12nM)	4	0,0006	***	Two-way ANOVA
		WT vs. R-CRIZ(6,25nM)	4	<0,0001	***	Two-way ANOVA
		WT vs. R-CRIZ(12,5nM)	4	<0,0001	***	Two-way ANOVA
		WT vs. R-CRIZ(25nM)	4	<0,0001	***	Two-way ANOVA
		WT vs. R-CRIZ(50nM)	4	<0,0001	***	Two-way ANOVA
		WT vs. R-CRIZ(100nM)	4	<0,0001	***	Two-way ANOVA

T-TEST was performed with Microsoft Excel Software; One-way and Two-way ANOVA (followed by Bonferroni ‘s multiple comparison test) were performed with GraphPad software.
