# Supplementary material for: miR‐205 mediates adaptive resistance to MET inhibition via ERRFI1 targeting and raised EGFR signaling
Source: EMBO Mol Med. 2018 Jul 24;10(9):e8746. doi: 10.15252/emmm.201708746 (PMC6127885; doi:10.15252/emmm.201708746)
Supplement: Supplementary file 2 — Expanded View Figures PDF [file EMMM-10-e8746-s002.pdf]

Expanded View Figures

Figure EV1. MiR-205 expression is increased in MET-TKI-resistant cells.

A Hierarchical clustering of miRNA expression profile reveals a cell line-specific pattern of expression. MiRNAs were clustered on the basis of the cell line of origin. Each row represents a miRNA, expressed as ΔCt calculated respect to the internal control (RNU48); each column represents a cell line. For each miRNA, units (−1 to +1) represent the ΔCt values against the median of the values in all the samples. Red and green colors denote higher or lower expression levels of the miRNA (median-centered), respectively.

B–F *MiR-205* expression was evaluated by RT–qPCR in EBC-1 (B), GTL16 (C), SG16 (D), KATO II (E), and SNU-5 (F) wt and resistant (R-) cells. As shown, *miR-205* was over-expressed in resistant versus wt cells. $n = 3$ per condition.

Data information: Average ± SD. Asterisks: ***$P < 0.001$; **$P < 0.01$; one-way ANOVA (B, C) and two-tailed t-test (D–F).

© 2018 The Authors

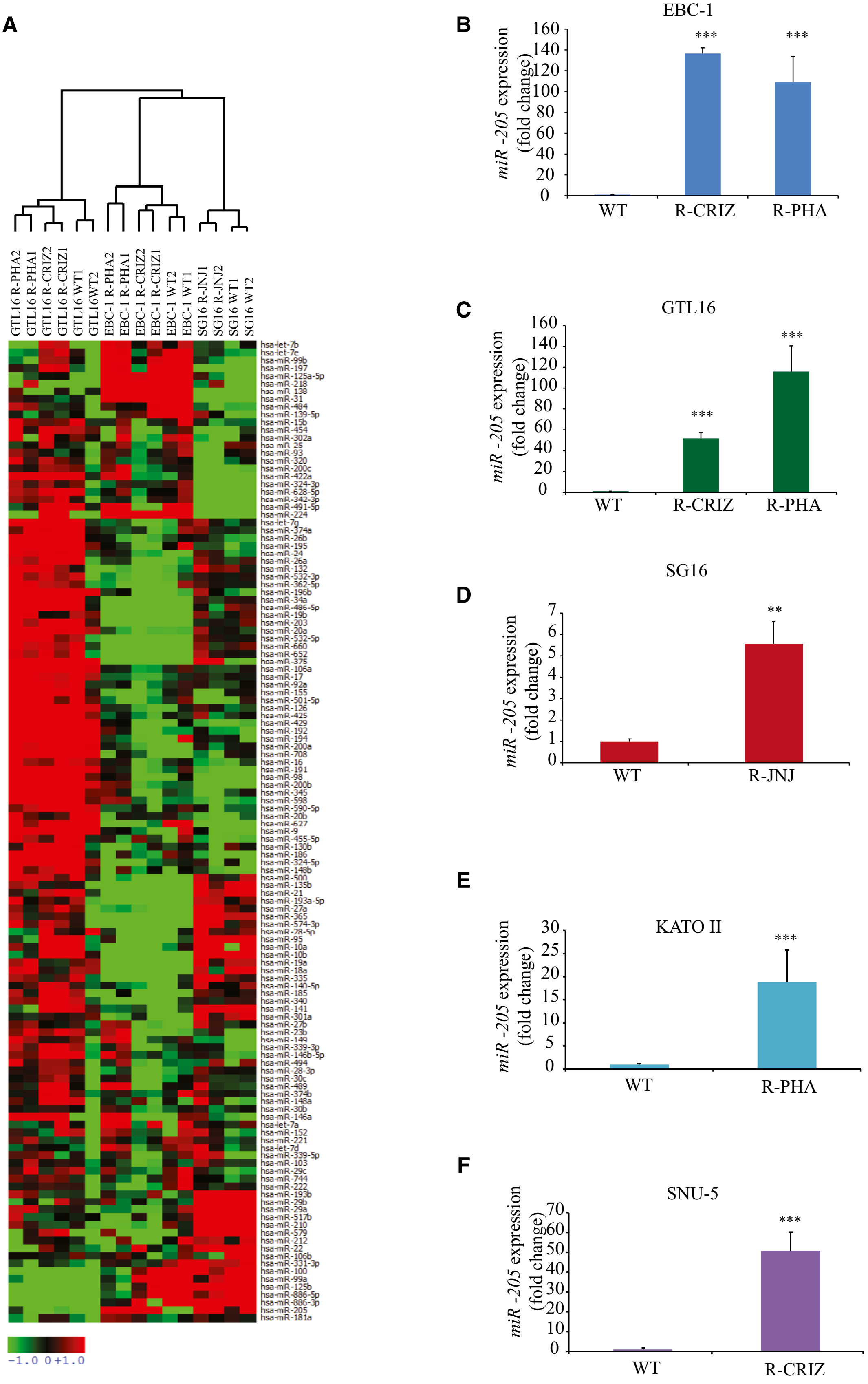

Figure EV1.

 © 2018 The Authors

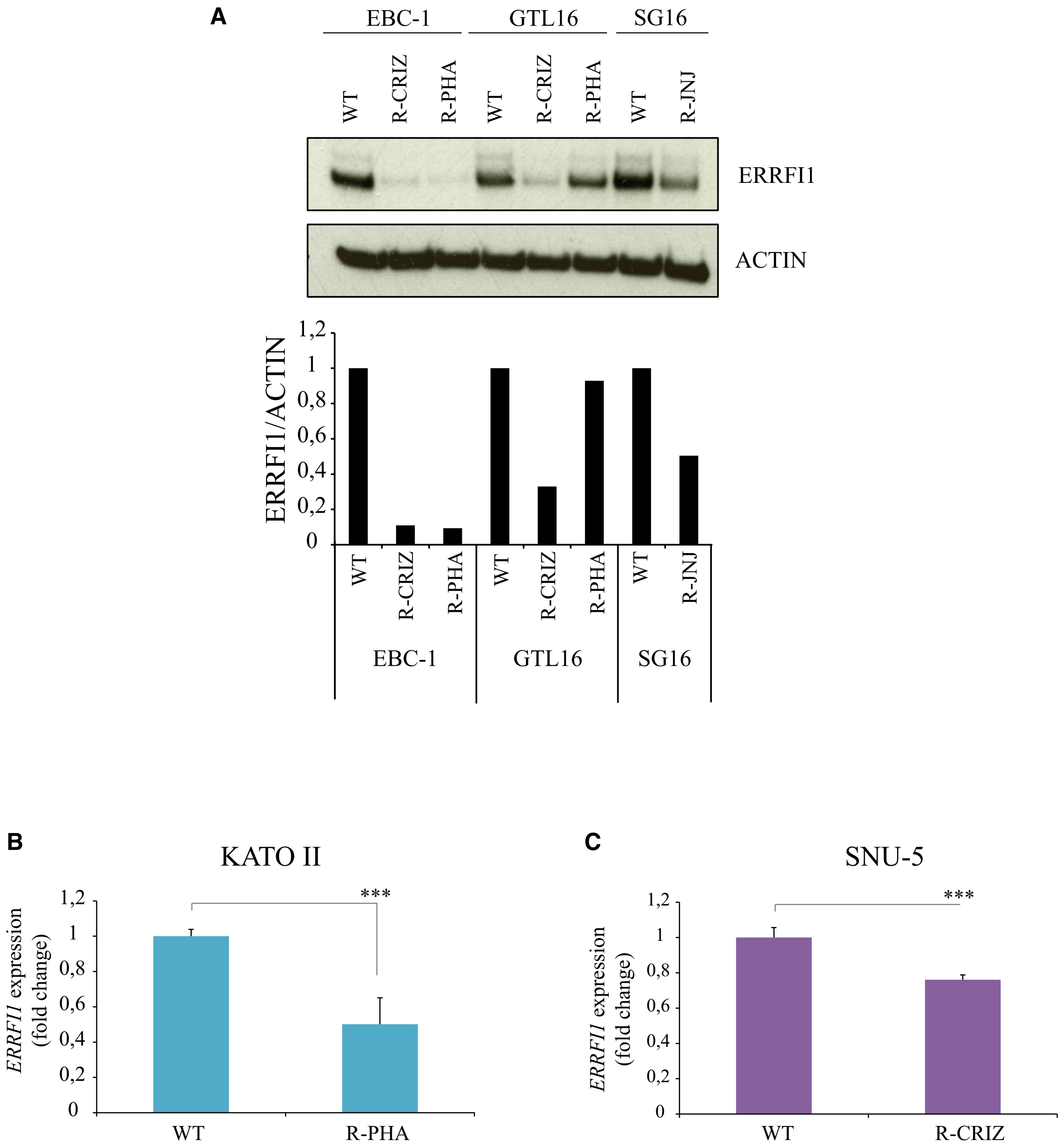

Figure EV2. ERRFI1 expression is decreased in MET-TKI-resistant cells.

A ERRFI1 expression was evaluated by WB in EBC-1, GTL16, and SG16 wt and resistant (R-) cells. ERRFI1 expression was strongly reduced in resistant cells compared to wt, with the exception of GTL16 R-PHA. Actin was used as loading control. Column chart shows the ERRFI1/Actin band quantification obtained by ImageJ software.

B, C *ERRFI1* expression was evaluated by RT–qPCR in KATO II (B) and SNU-5 wt and resistant (R-) cells. As shown, ERRFI1 was downregulated in resistant versus wt cells. $n = 3$ per condition.

Data information: (B, C) Average ± SD. ***$P < 0.001$; **$P < 0.01$, two-tailed t-test.
Source data are available online for this figure.

© 2018 The Authors

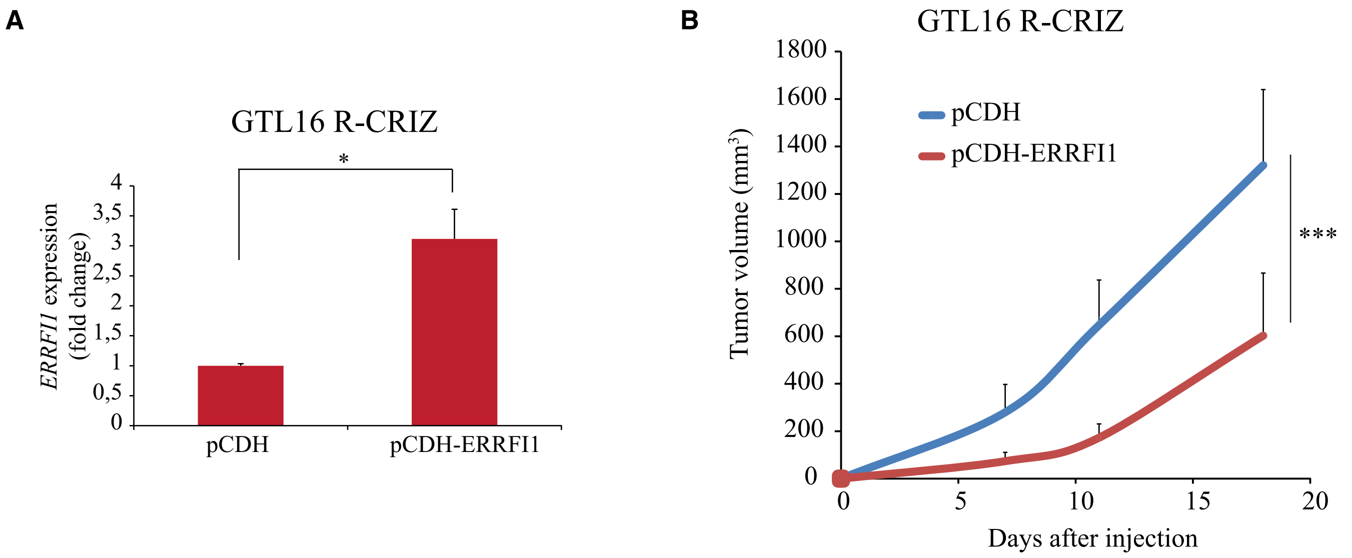

Figure EV3. ERRFI1 expression in GTL16 R-CRIZ cells partially reverts resistance to crizotinib treatment.

A ERRFI1 expression was evaluated by RT–qPCR in GTL16 R-CRIZ cells transduced with ERRFI1 (pCDH-ERRFI1) or the control vector (pCDH). $n = 3$ per condition.
B 10^6 GTL16 R-CRIZ cells transduced with ERRFI1 (pCDH-ERRFI1) or the control vector (pCDH) were subcutaneously injected in NOD/SCID mice. Mice were treated with crizotinib (25 mg/kg), and tumor volume was monitored at the indicated times for 18 days. Tumors generated by GTL16 R-CRIZ cells transduced with pCDH-ERRFI1 were significantly smaller compared to control cells, meaning that re-expression of ERRFI1 was able to partially revert resistance. $n = 6$ per condition.

Data information: Average ± SD. (A) *$P < 0.05$; two-tailed t-test. (B) ***$P < 0.001$, two-way ANOVA, Bonferroni's multiple comparisons test.

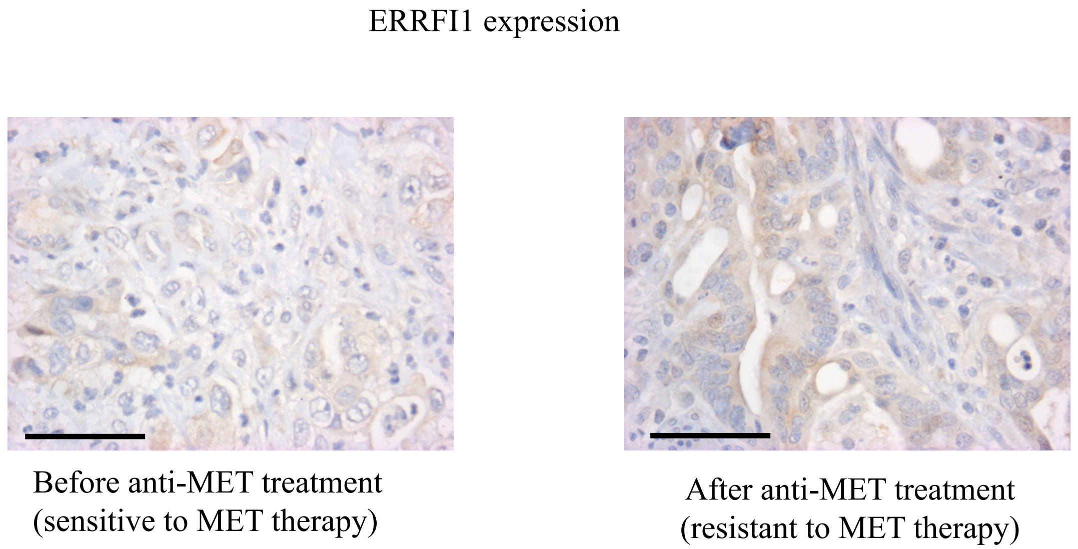

Figure EV4. ERRFI1 expression is not changed upon onset of acquired resistance to MET-TKIs in a metastatic colorectal cancer patient (Patient #1).

Immunohistochemical analysis of ERRFI1 expression in a metastatic *BRAF*-mutated colorectal cancer patient becomes resistant to anti-EGFR plus BRAF inhibitor treatment (panitumumab + vemurafenib) as consequence of *MET* amplification (Pietrantonio *et al*, 2016; Oddo *et al*, 2017). The patient was then treated with vemurafenib plus crizotinib but, after an initial partial response, progressed. No difference in ERRFI1 expression was detected between the two samples obtained before (left) and after (right) BRAF and MET combined inhibition. Scale bar = 62.5 µm.

 © 2018 The Authors

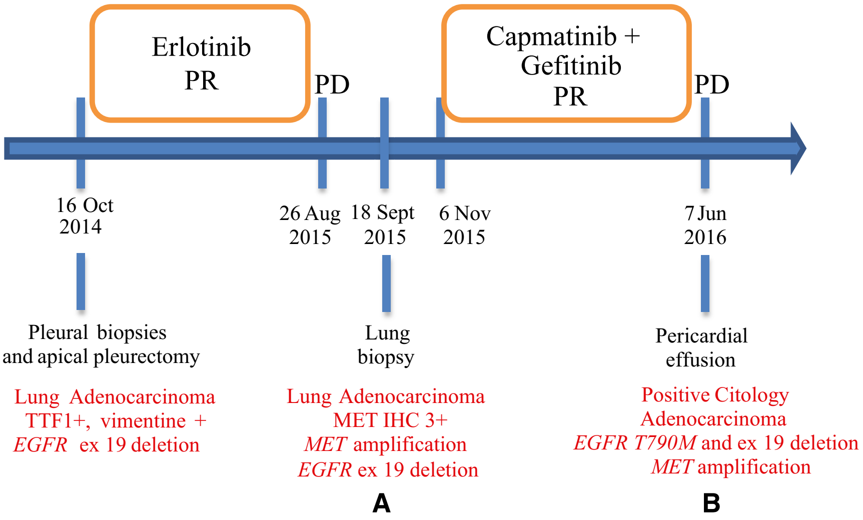

Figure EV5. Scheme of the clinical course of patient #2 affected by non-small-cell lung cancer.

Boxes indicate periods of administration of the indicated agents. Blue vertical lines indicate dates of tumor specimen acquisition (from surgical procedure or biopsy) or of tumor assessment (CT scan or FDG-PET/CT scan). Available histological and molecular characterizations are reported in red. PR = partial response; PD = progressive disease. (a) and (b) correspond to the samples shown in Fig 4.

© 2018 The Authors
