# Supplementary figures and images for: miR‐205 mediates adaptive resistance to MET inhibition via ERRFI1 targeting and raised EGFR signaling
Source: EMBO Mol Med. 2018 Jul 24;10(9):e8746. doi: 10.15252/emmm.201708746 (PMC6127885; doi:10.15252/emmm.201708746)

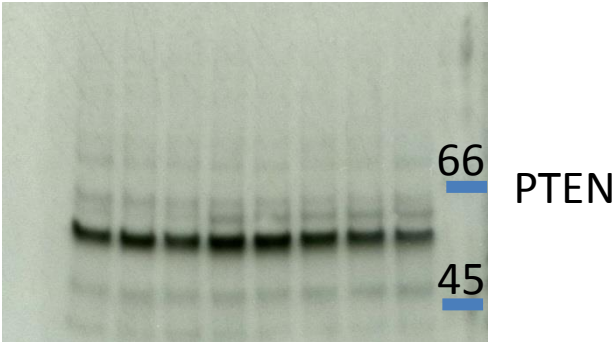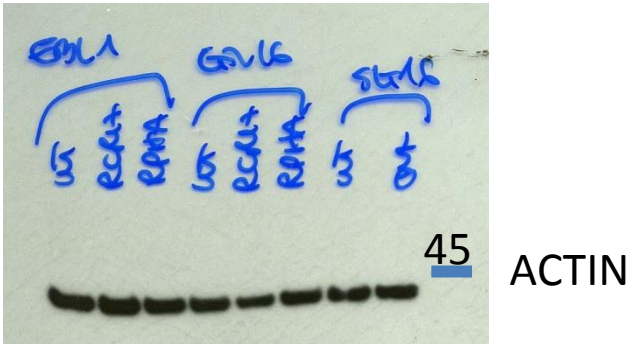

Supplement: Supplementary file 3 — Source Data for Expanded View and Appendix [file EMMM-10-e8746-s003.zip › EVandappendixsd/EV-and-appendix-sd/EMM-2017-08746-V2_Source_Data_Appendix_Figure_S5_UPDATED.pdf]

Referred to FIGURE EV2

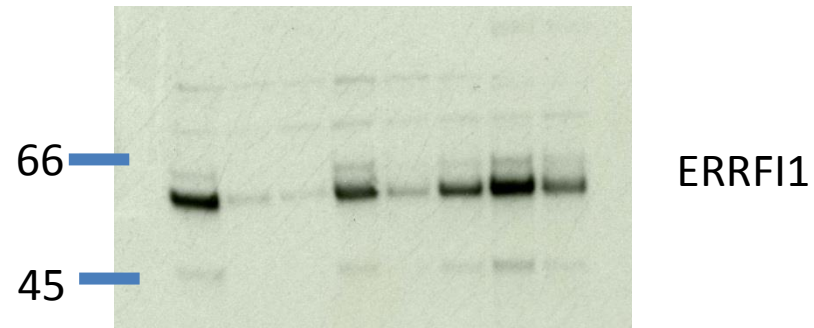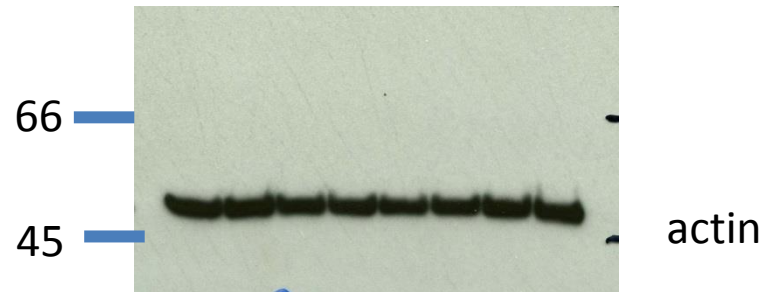

Supplement: Supplementary file 3 — Source Data for Expanded View and Appendix [file EMMM-10-e8746-s003.zip › EVandappendixsd/EV-and-appendix-sd/EMM-2017-08746-V2_Source_Data_Figure_EV2_UPDATED-3.pdf]

Referred to APPENDIX FIGURE S7

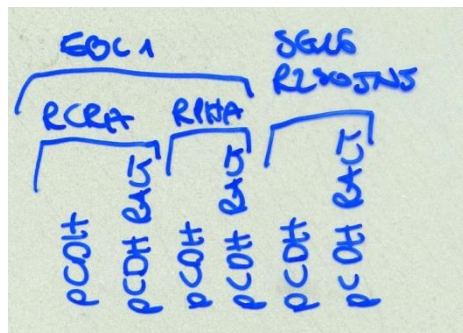

GTL16

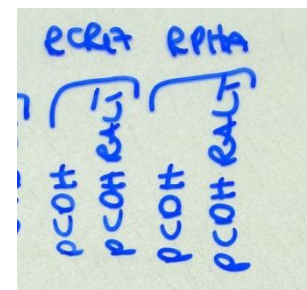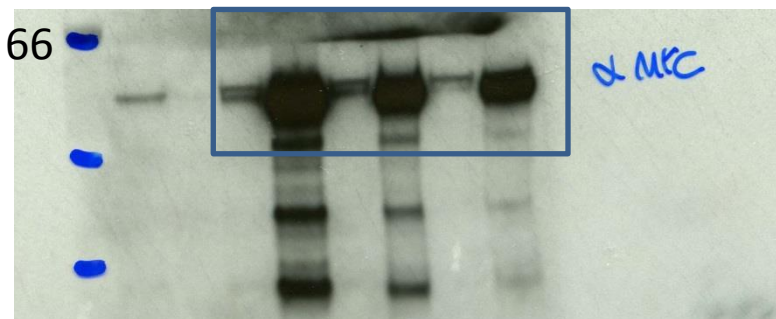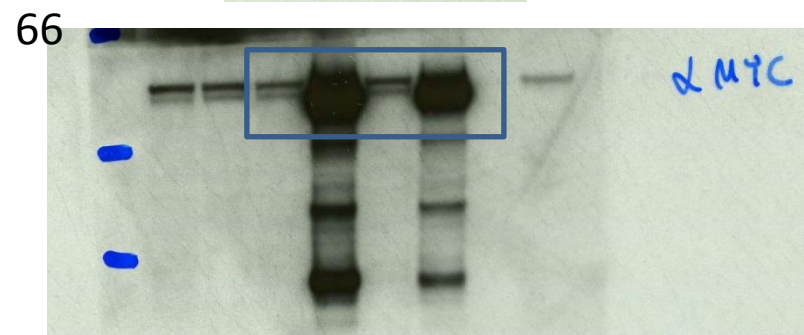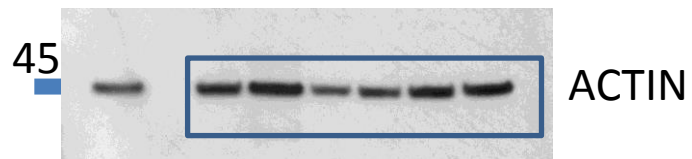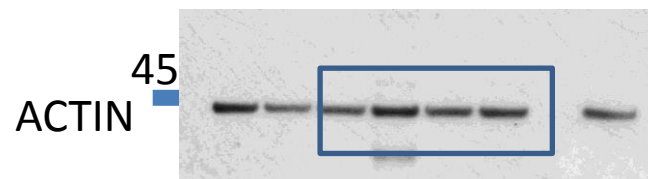

Supplement: Supplementary file 3 — Source Data for Expanded View and Appendix [file EMMM-10-e8746-s003.zip › EVandappendixsd/EV-and-appendix-sd/EMM-2017-08746-V2_Source_Data_for_Appendix_Figure_S7_UPDATED-2.pdf]

A549

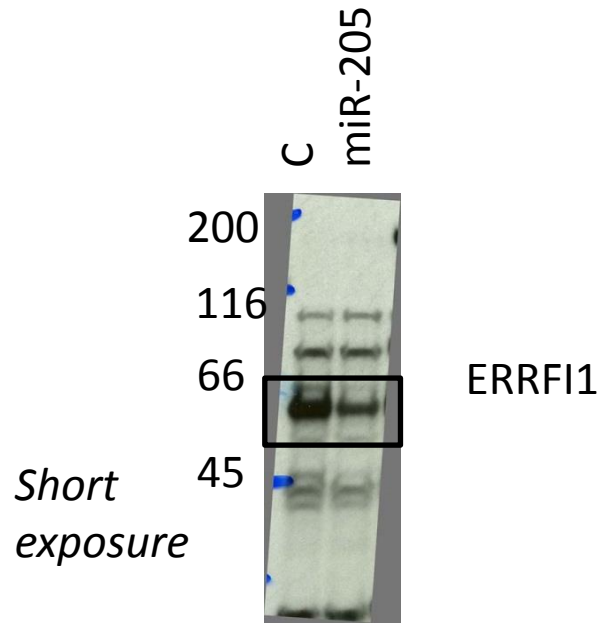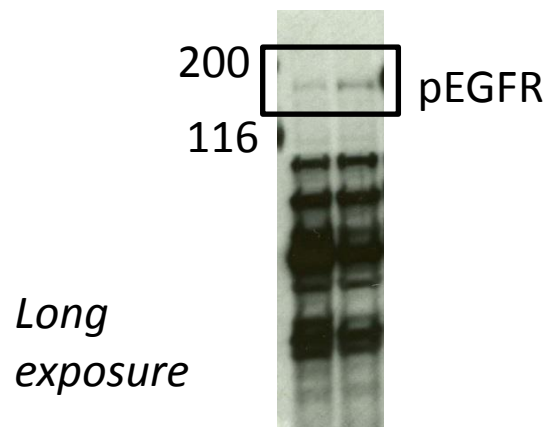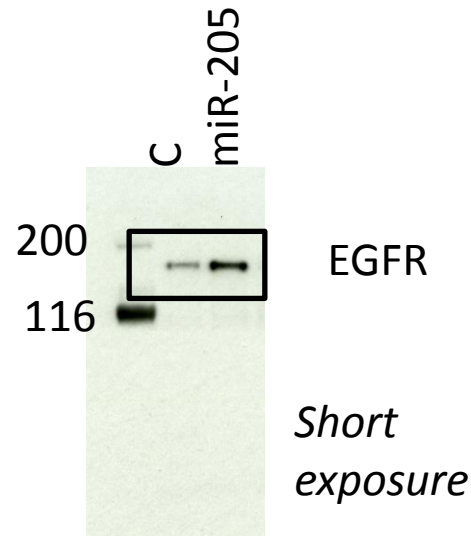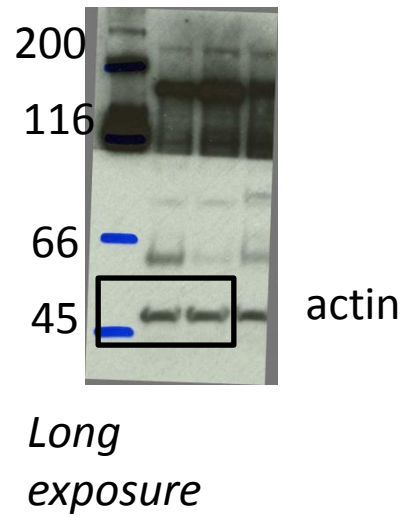

Supplement: Supplementary file 3 — Source Data for Expanded View and Appendix [file EMMM-10-e8746-s003.zip › EVandappendixsd/EV-and-appendix-sd/EMM-2017-08746-V2_Source_Data_for_Appendix_Figure_S8.pdf]

PANEL D

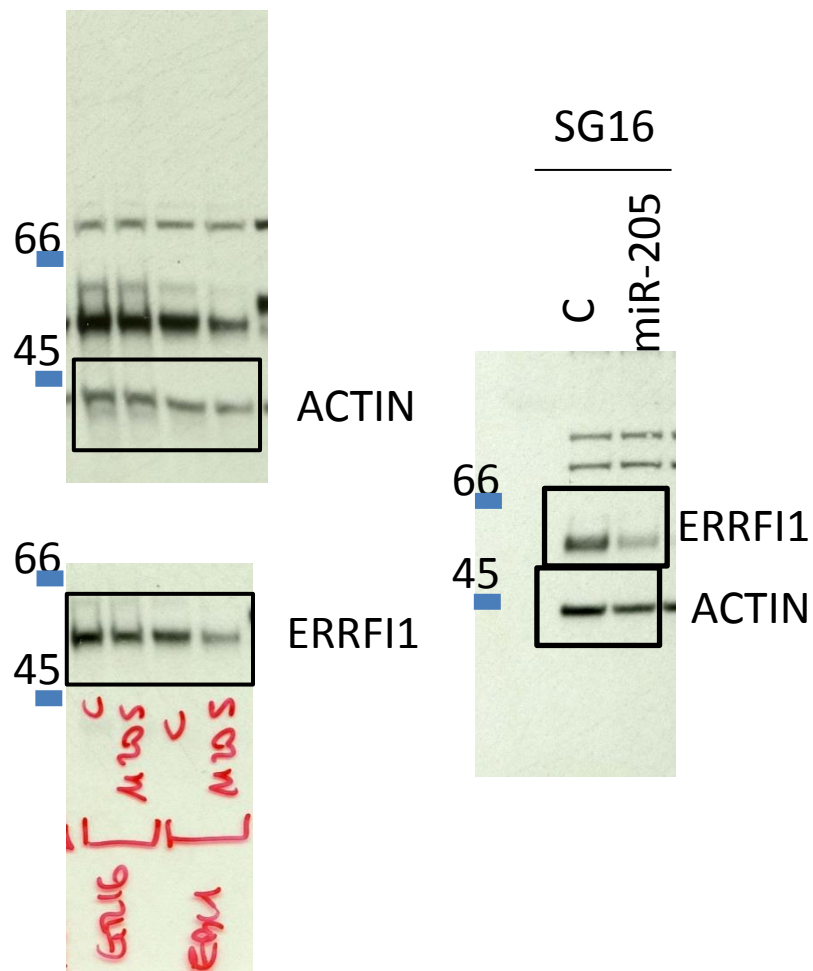

PANEL G

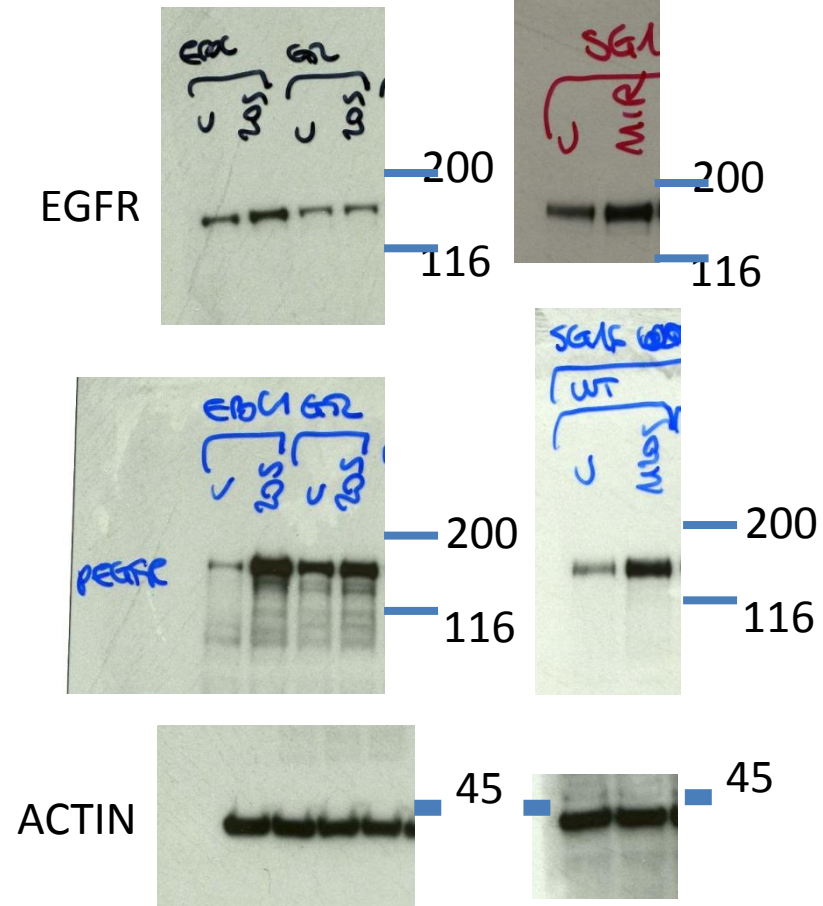

Supplement: Supplementary file 6 — Source Data for Figure 3 [file EMMM-10-e8746-s005.pdf]
