# Supplementary material for: miR‐205 mediates adaptive resistance to MET inhibition via ERRFI1 targeting and raised EGFR signaling
Source: EMBO Mol Med. 2018 Jul 24;10(9):e8746. doi: 10.15252/emmm.201708746 (PMC6127885; doi:10.15252/emmm.201708746)
Supplement: Supplementary file 5 — Source Data for Figure 2 [file EMMM-10-e8746-s004.pdf]

EBC-1

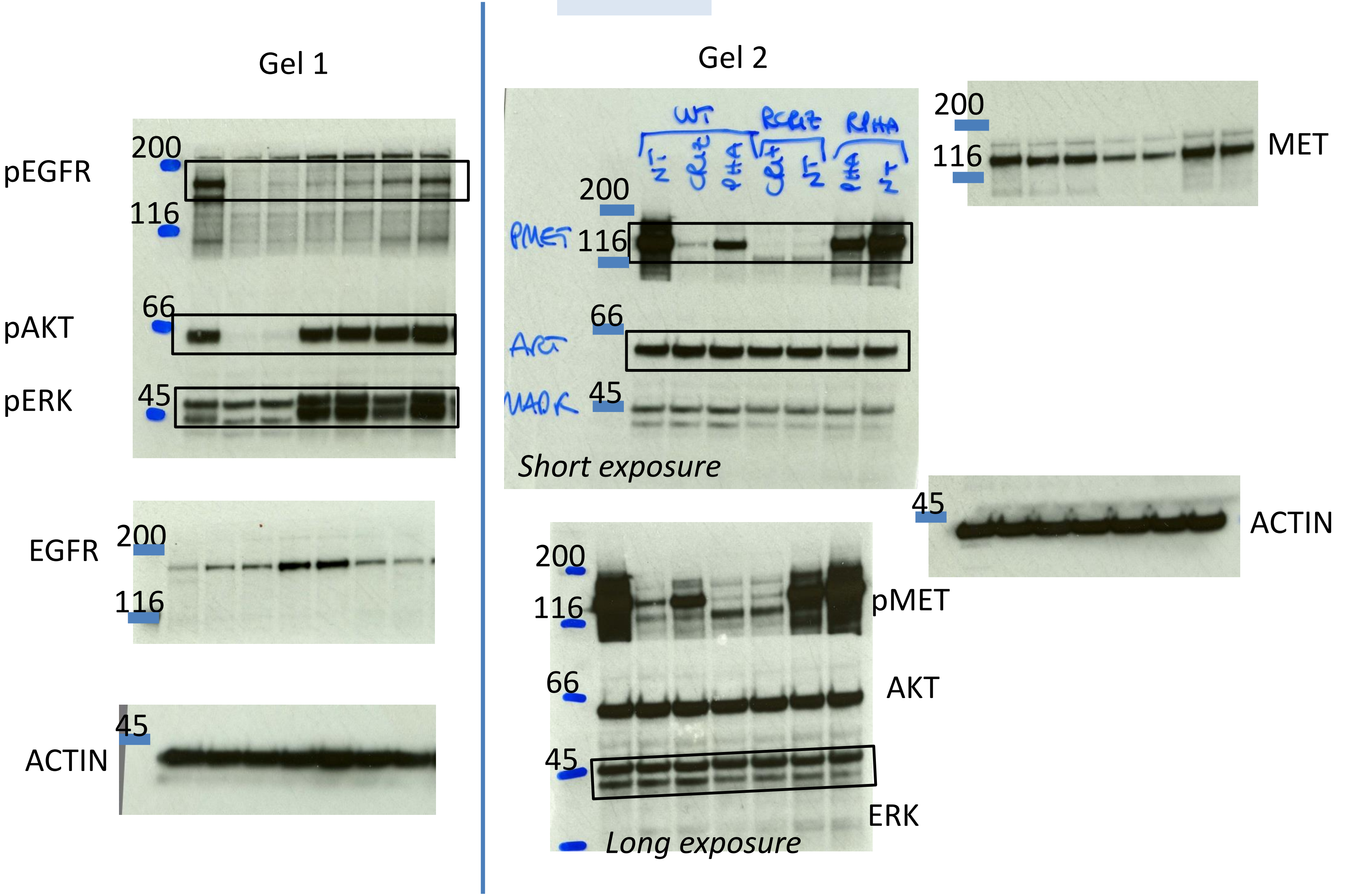

Gel 1
pEGFR
200
116
pAKT
66
pERK
45
EGFR
200
116
ACTIN
45
Gel 2
WT
200
pMET
116
66
45
Short exposure
200
116
MET
45
ACTIN
200
116
pMET
66
AKT
45
ERK
Long exposure

GTL16

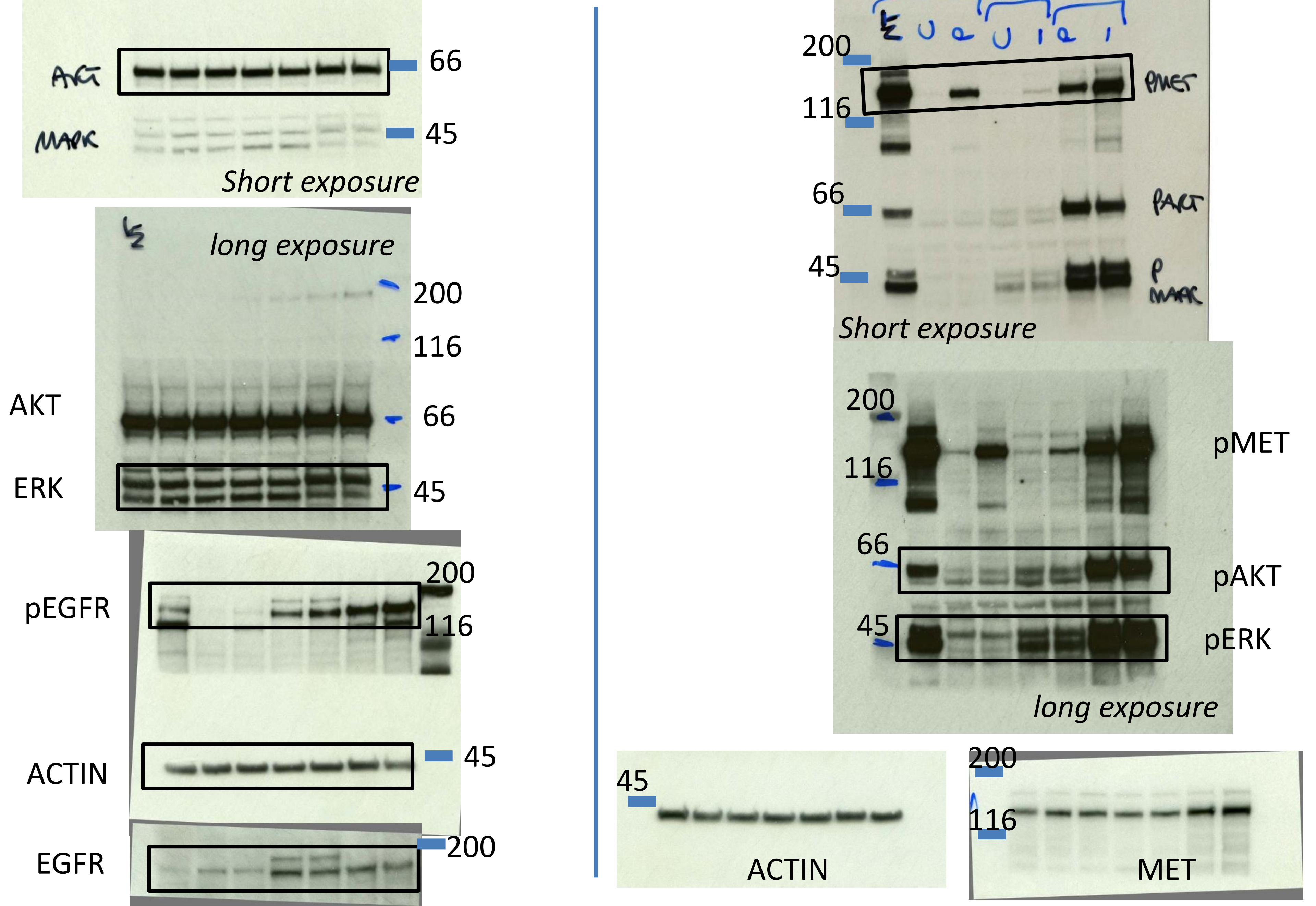

Gel 1
66
45
Short exposure
long exposure
200
116
66
45
AKT
ERK
pEGFR
200
116
ACTIN
45
EGFR
200
Gel 2
200
116
66
45
Short exposure
200
116
66
45
pMET
pAKT
pERK
long exposure
45
ACTIN
200
116
MET

SG16

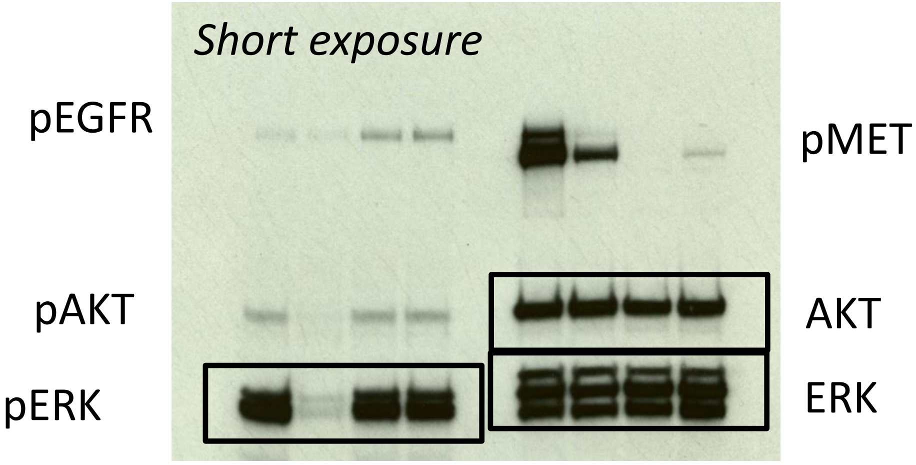
Short exposure
pEGFR
pMET
pAKT
AKT
pERK
ERK

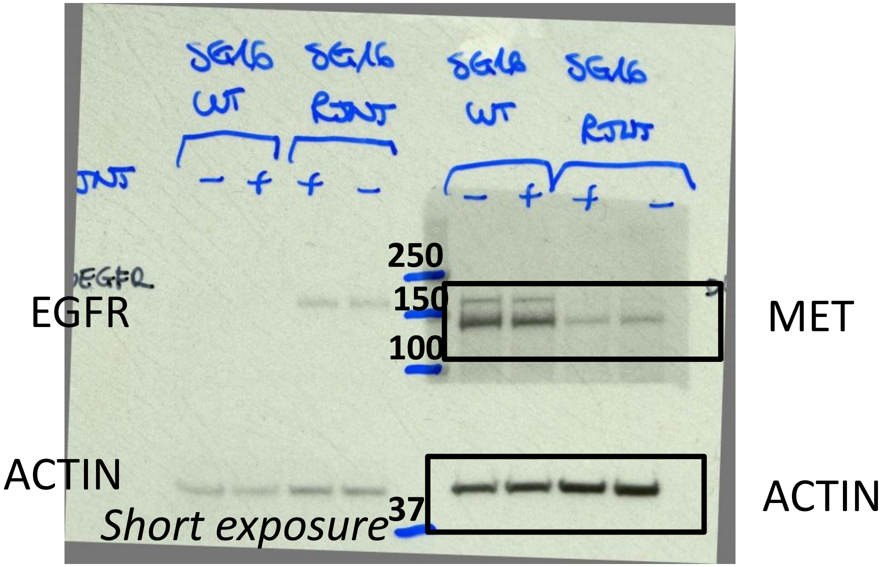
EGFR
MET
ACTIN
ACTIN
250
150
100
37
Short exposure

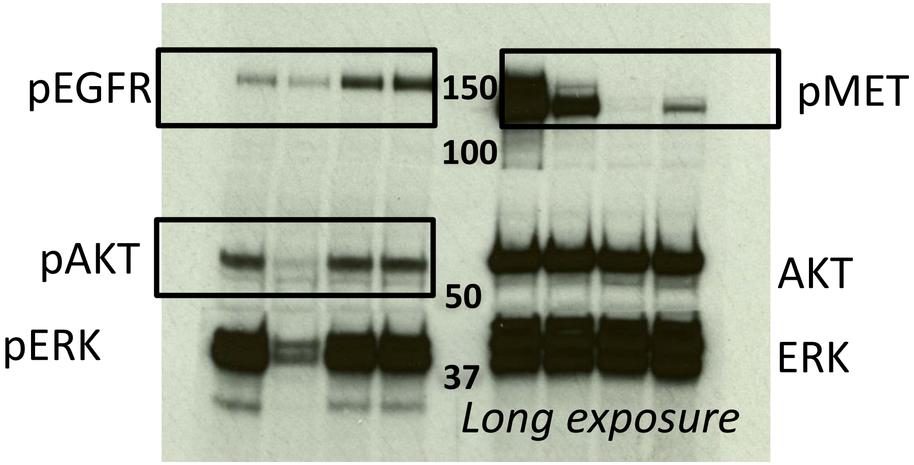
pEGFR
pMET
150
100
pAKT
AKT
50
pERK
ERK
37
Long exposure

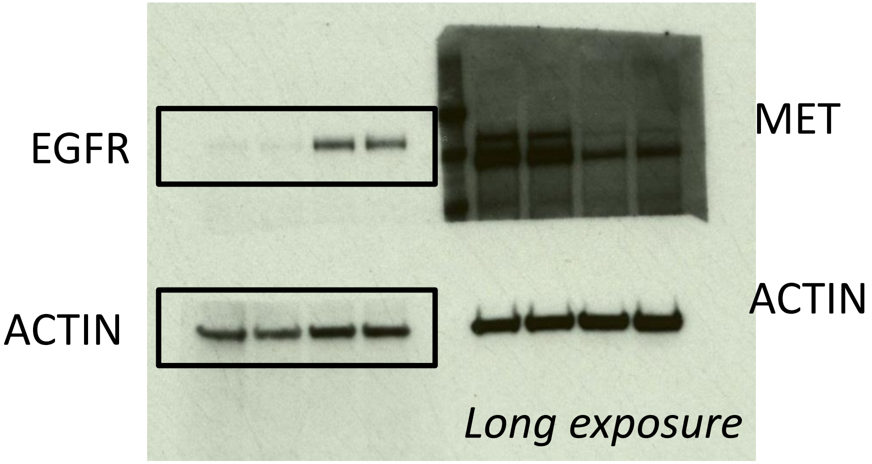
MET
EGFR
ACTIN
ACTIN
Long exposure
